# The role of m^6^A demethylase FTO in chemotherapy resistance mediating acute myeloid leukemia relapse

**DOI:** 10.1038/s41420-023-01505-y

**Published:** 2023-07-05

**Authors:** Zhi-Wei Zhang, Xiao-Su Zhao, Huidong Guo, Xiao-Jun Huang

**Affiliations:** 1 Peking University People’s Hospital & Peking University Institute of Hematology, National Clinical Research Center for Hematologic Disease, Beijing Key Laboratory of Hematopoietic Stem Cell Transplantation, Peking University, 100044 Beijing, China; 2 Peking-Tsinghua Center for Life Sciences, School of Life Sciences, Peking University, 100044 Beijing, China

**Keywords:** Acute myeloid leukaemia, Epigenomics

## Abstract

Acute myeloid leukemia (AML) is the most common hematopoietic malignancies, and chemotherapy resistance is one of the main causes of relapse. Because of lower survival rate for patients with relapse, it is pivotal to identify etiological factors responsible for chemo-resistance. In this work, direct MeRIP-seq analysis of sequential samples at stage of complete remission (CR) and relapse identifies that dysregulated N6-methyladenosine (m^6^A) methylation is involved in this progression, and hypomethylated RNAs are related to cell differentiation. m^6^A demethylase FTO is overexpressed in relapse samples, which enhances the drug resistance of AML cells in vivo and in vitro. In addition, FTO knockdown cells exhibit stronger capacity of differentiation towards granules and myeloid lineages after cytosine arabinoside (Ara-C) treatment. Mechanistically, *FOXO3* is identified as a downstream target of FTO, the hypomethylation of *FOXO3* mRNA affects its RNA degradation and further reduces its own expression, which ultimately result in attenuated cell differentiation. Collectively, these results demonstrate that FTO-m^6^A-FOXO3 is the main regulatory axis to affect the chemotherapy resistance of AML cells and FTO is a potential therapeutic target of chemotherapy resistance in AML.

## Introduction

Acute myeloid leukemia (AML) is one of the most common hematopoietic malignancies, characterized with high heterogeneity, abnormal clonal expansion and differentiation block of progenitor cells [1], which is a serious risk to human health.

Currently, the main treatment for AML is still chemotherapy. Although most of patients can achieve complete remission (CR) undergoing conventional chemotherapy [2], 5-year survival rates for younger (aged <60 years) and older patients (aged >60 years) reached only 40% and <20% respectively, indicates most of them are diagnosed refractory disease or relapse [3–8]. Because of limited treatment options, relapse is the leading cause of death in AML [9–12]. The main factor of relapse is chemo-resistance, so it is pivotal to identify etiological factors responsible for chemo-resistance, which relies on comprehensive understanding of the molecular mechanism underlying drug response. In the prevailing view, gene mutation, abnormal regulation of RNA and protein may lead to drug resistance. For example, some gene mutations occurred in autophagy signal pathway and drug resistance related protein, abnormal expression of mRNAs and microRNAs will lead to drug resistance in AML patients [3, 13–15].

Epigenetics has been proved to play an important role in physiological and pathological processes. RNA N6-methyladenosine (m^6^A) is the most abundant internal modification in mRNAs, including the hematopoietic system development and the maintenance of hematopoietic stem cell function [16–21]. Under the regulation of writers, erasers and readers, m^6^A plays important roles in regulating RNA fate, such as RNA degradation [22], nuclear export [23] and so on [24, 25]. Emerging evidence has shown many types of cancers are characterized by abnormal m^6^A modification due to dysfunctional m^6^A regulators, especially in AML [26]. It has been reported that m^6^A writers (METTL3, METTL14, METTL16), erasers (FTO, ALKBH5) and readers (IGF2BP family, YTHDC1) are all involved in regulating AML progression [27–31]. Thus, intervention of key regulators of m^6^A modification has been recognized as a potential therapeutic approach for cancer therapy [32–37].

FTO is one of m^6^A demethylases and closely related to fat-mass and obesity [38–40]. It has been reported that FTO decreased the drug resistance of melanoma and cervical squamous cell carcinoma through mRNA demethylation [41, 42]. However, there is no evidence of the relationship between m^6^A modification and chemotherapy resistance in AML. Thus, investigating the m^6^A regulation in chemotherapy resistance AML may provide more comprehensive insights of recurrence after chemotherapy, and help develop more effective targeted therapies to treat AML.

In the present study, we sought to investigate whether dysregulated RNA m^6^A methylation involves in AML cell resistant to chemotherapy and explore the underlying molecular mechanism. Our results indicate that the elevated expression of FTO plays a critical role in chemotherapy resistance. Combined MeRIP-Seq analysis and functional experiments, we found *FOXO3* as a downstream target of FTO. The results suggested that FTO decreased the m^6^A modification of *FOXO3* and enhanced its mRNA degradation rate. In summary, our study provides insights into the multiple molecular mechanisms of chemotherapy resistance in AML, which leads to the recurrence.

## Results

### Abnormal m^6^A modification is found in relapsed samples

**Fig. 1:**
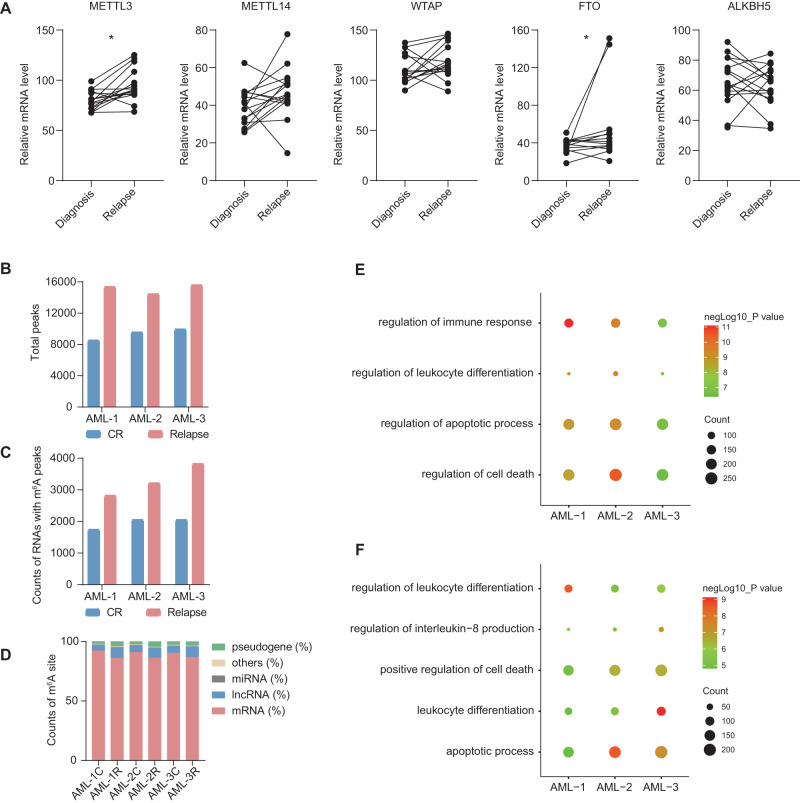
Transcriptome-wide RNA m^6^A methylation analysis of relapse and control samples. **A** RNA expression level comparison of core m^6^A methyltransferases and demethylases between sequential diagnosis (*n* = 16) and relapse samples (*n* = 16) from a public dataset. *P*-values were calculated using Wilcoxon test, and *P* < 0.05 was considered as statistically significant. *P < 0.05. **B** Bar graph showing the total peaks identified in three pairs of sequential samples. **C** The statistics of the counts of RNAs with m^6^A peak in three pairs of sequential samples. **D** Bar graph showing the m^6^A-modified RNA classification. C represents CR and R represents relapse **E**, **F** Gene ontology (GO) analysis of the RNAs with m^6^A peaks in CR (**E**) and relapse (**F**) samples.

In order to investigate the impact of dysregulated RNA m^6^A methylation in AML relapse, we first analyzed the alterations of m^6^A regulators (10 writers, 2 erasers and 12 readers) between diagnosis and relapse samples based on published article [43]. The expression of m^6^A regulatory factors changed between diagnosis and relapse of patients, among which METTL3 and FTO were both increased at relapse (Fig. 1A and Fig. S1A).

Previous studies reported that the resistance of tumor cells to chemotherapy drugs is one of the important reasons for recurrence [44]. Considering the both high AML cell burden at diagnosis and relapse, we picked sequential samples underwent CR after chemotherapy and eventually relapse for subsequent experiments (Table S1). To analyze the change of m^6^A methylation, we performed MeRIP-seq by using sequential samples at CR and relapse after chemotherapy, respectively (Table S2). In line with previous findings, “UGGAC” was the most conserved motif in the m^6^A peaks identified from all samples (Fig. S1B), which were unevenly distributed across the whole transcripts as previously reported (Fig. S1C, S1D).

Under standard identification conditions, a higher number of both m^6^A peaks and RNAs with m^6^A modifications were identified in the recurrent samples (Fig. 1B, C), which were mainly distributed on mRNA (Fig. 1D). Interestingly, increased m^6^A modification on lncRNA during relapse suggests that lncRNA also involved in regulating AML relapse (Fig. 1D). Next, Gene ontology (GO) analysis showed that the genes with m^6^A peaks in CR and relapse samples was mainly enriched in cell differentiation, cell death and apoptosis related pathways (Fig. 1E, F and Table S3), indicates that altered chemotherapy resistance in AML cells may be related to these functions. Taken together, the above results suggest that dysregulated m^6^A modification plays important roles in AML relapse.

### Hypomethylated genes at recurrence are enriched in cell differentiation

**Fig. 2:**
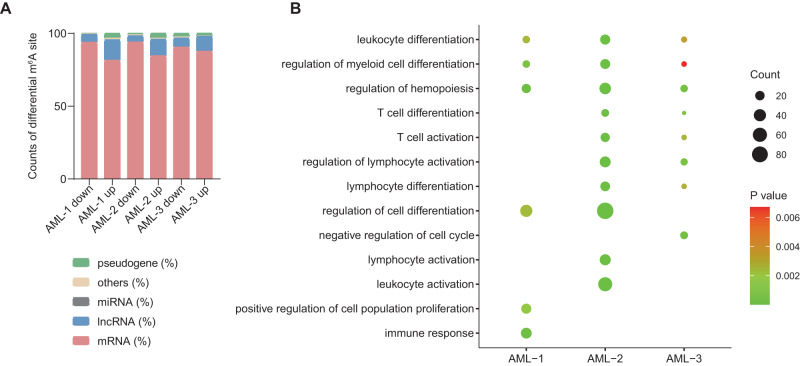
Analysis of differential N6-methyladenosine (m^6^A) methylation in sequential samples. **A** Bar graph showing the m^6^A-modified RNA classification with hypomethylated (down) and hypermethylated (up) sites. **B** GO analysis of the hypomethylated RNAs in relapse samples.

Based on the dysregulation of m^6^A in AML samples during recurrence, we further analyzed differentially methylated m^6^A modification with three pairs of sequential samples (Table S4), and the results were in consistent with previous (Fig. S2A, S2C). In addition to that, we also found the proportion of differentially methylated peaks also increased in lncRNAs (Fig. 2A), indicates that m^6^A modification on lncRNA may also be involved in the regulation of AML recurrence.

To investigate the functions affected by abnormal m^6^A modification, we next performed GO analysis of differentially methylated genes. As Fig. 2B showed that m^6^A-hypomethylated genes mainly related to cell differentiation, hematopoietic regulation and cell activation, among which “leukocyte differentiation”, “regulation of myeloid cell differentiation” and “regulation of hemopoiesis” these three pathways were enriched in all three paired samples (Fig. 2B and Table S5), while the m^6^A-hypermethylated genes were not found co-exist pathways (Fig. S2D and Table S5). These results indicate that hypomethylated genes related to cell differentiation play roles in AML relapse.

### FTO suppression reduces the chemo-resistance of AML cells through cell differentiation

**Fig. 3:**
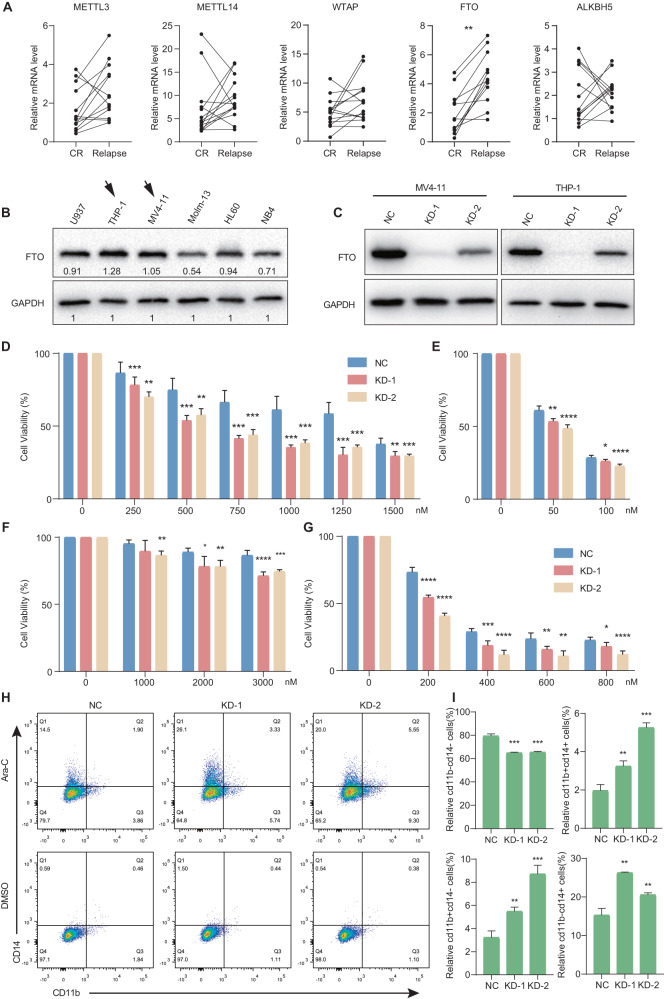
The effects of FTO on AML cell chemotherapy resistance in vitro. **A** Comparison of RNA expression between complete remission (CR) and relapse samples. **B** Western blot analysis showing the expression level of FTO in six AML cell lines. Highly FTO expressed cell line were marked by black arrows. **C** Western blot analysis confirming the effects of FTO knockdown in MV4-11 and THP-1 cells. **D**, **E** Cell viability assay showing the effects of FTO on chemotherapy resistance with Ara-C (**D**) or DOX (**E**) treatment in MV4-11 cells. **F**, **G** Cell viability assay showing the effects of FTO on chemotherapy resistance with Ara-C (**F**) or DOX (**G**) treatment in THP-1 cells. **H**, **I** Flow cytometry showing the regulation of FTO to cell differentiation with Ara-C treatment, DMSO was used as vehicle (**H**). The statistical analysis was shown in **I**. Representative results from three replicates were shown here. *P*-values were calculated using Two-tailed Student’s *t*-test, and *P* < 0.05 was considered as statistically significant. *P < 0.05, **P < 0.01, ***P < 0.001 and ****P < 0.0001.

Based on previous data, we compared the expression level of core m^6^A methylases and demethylases in sequential samples from 14 patients. Interestingly, we found that FTO was highly expressed in relapse (after chemotherapy) samples (Fig. 3A), which suggests that high expression of FTO may regulate the chemo-resistance and relapse of AML. Therefore, we explored the effect of FTO on chemotherapy resistance of AML cells. Cell viability assay was performed using two human AML cell lines (MV4-11 and THP-1) with high FTO expression (Fig. 3B). In the treatment of cytarabine (Ara-C) or doxorubicin (DOX), FTO knockdown (KD) (Fig. 3C) resulted in a significant reduction of chemotherapy resistance in both cell lines (Fig. 3D–G). Combined the regulation of FTO to chemo-resistance with GO analysis of hypomethylated genes, we hypothesized that overexpression of FTO leads to chemotherapy resistance through abnormal cell differentiation.

To verify this hypothesis, we performed flow cytometry to identify the effect of FTO on cell differentiation after Ara-C treatment. FTO knockdown cells exhibit stronger capacity of differentiation towards granules and myeloid lineages (Fig. 3H, I). Taken together, the above results indicate that FTO can alter the chemotherapy resistance of AML cells in terms of mediating their differentiation process.

For further verification, we performed functional experiments using Ara-C resistant (Resis) cells. Representative AML cell line MV4-11 was initially exposed to increasing concentrations of the chemotherapeutic drug Ara-C, until they could grow in medium containing high dose of drug (Fig. S3A). The cell viability results showed that knocking down FTO in Ara-C resistant cells could partially reduce drug resistance (Fig. S3B). In addition, drug-resistant cells showed increased expression of CD11b and CD14 (Fig. S3C), and exhibited a weak tendency of continued differentiation after FTO knockdown (Fig. S3D, S3E). Compared the FTO function between sensitive and Ara-C resistant cells, these results suggested that although FTO plays roles in drug resistance of AML cells, inhibition of FTO expression has little significance in delaying the AML progression in drug-resistant cells.

### The knockdown of FTO decreases chemotherapy resistance in vivo

**Fig. 4:**
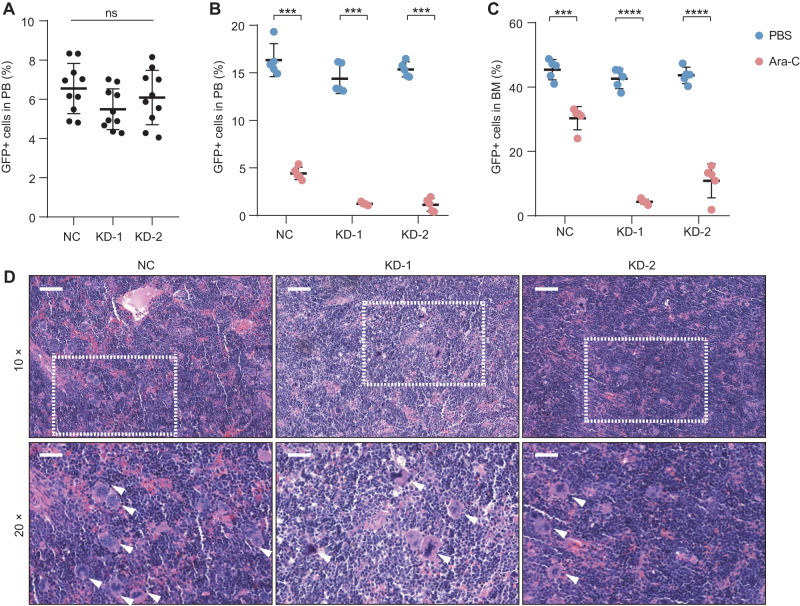
The effects of FTO on AML cell chemotherapy resistance in vivo. **A** Flow cytometry to detect the percentage of GFP+ cells in peripheral blood (PB) before Ara-C treatment. **B** Flow cytometry to detect the percentage of GFP+ cells in peripheral blood after Ara-C treatment. **C** Flow cytometry to detect the percentage of GFP+ cells in bone marrow after Ara-C treatment. **D** Representative images of tumor cell infiltration in spleen. The tumor cells were marked by white arrows. Scale bars represent 100 μm (upper panel) and 50 μm (lower panel), respectively, and the regions of lower panel were marked with white dotted line. *P-*values were calculated using Two-tailed Student’s *t*-test, and *P* < 0.05 was considered as statistically significant. ***P < 0.001 and ****P < 0.0001, ns: not significant.

To verify the role of FTO in mediating chemotherapy resistance in AML progression, we assessed the effect of FTO-knockdown in NPG mice with two regimens of drug administration: vehicle and Ara-C (100 mg/kg, every two days, intravenous injections). After confirming that the proportion of transplanted tumor cells (GFP+) in mice bone marrow (BM) was higher than that in peripheral blood (PB) through flow cytometry (supplementary Fig. S4A–S4C), we divided thirty mice into three groups randomly and injected tumor cells (negative control (NC), KD-1, KD-2) through tail vein. The mice were treated with Ara-C or PBS when the percentage of GFP+ cells in PB reached approximately 5% without weight change (Fig. 4A and Fig. S4D). Consistent with our previous results in vitro, knockdown of FTO significantly decreased the inhibitory effects of Ara-C treatment in vivo (Fig. 4B, C). Compared the spleen size between administration and control group, we obtained that the spleen size of control group was larger than administration group (Fig. S4E). Moreover, the tumor cell infiltration in NC was stronger than FTO knocked down in the spleen (Fig. 4D). In general, the results in vivo confirmed that the knockdown of FTO significantly decreased chemotherapy resistance.

### *FOXO3*, *CREBBP*, and *SIRPA* are potential targets of FTO

**Fig. 5:**
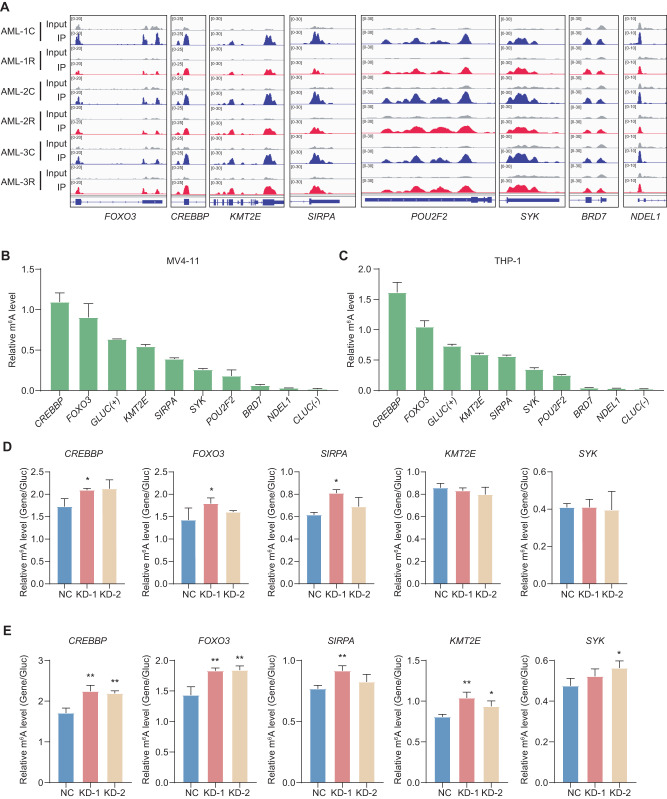
The effects of FTO knockdown on the m^6^A levels of indicated transcripts. **A** Genome browser tracks of m^6^A peaks in the selected transcripts in complete remission and relapse samples, C: complete remission; R: relapse. **B**, **C** m^6^A-IP-qPCR results showing the level of m^6^A modification in MV4-11 (**B**) and THP-1 (**C**) cell lines. *GLUC* represents positive control and *CLUC* represents negative control. **D**, **E** m^6^A-IP-qPCR results showing the effects of FTO knockdown on the m^6^A levels in MV4-11 (**D**) and THP-1 (**E**) cell lines of indicated transcripts. Representative results from three replicates were shown here. *P*-values were calculated using Two-tailed Student’s *t*-test, and *P* < 0.05 was considered as statistically significant. *P < 0.05, **P < 0.01.

To elucidate the molecular mechanism of FTO in regulating chemotherapy resistance of AML cells, we next searched for downstream targets of FTO, which were enriched in the pathways related to cell differentiation (Fig. 2B) and exhibited differential methylation levels in relapsed samples. We screened Integrative Genomics Viewer (IGV) profiles for hypomethylated genes associated with the cell differentiation obtained by MeRIP-seq (Fig. 5A). The m^6^A-IP-qPCR assay further verified that *CREBBP*, *FOXO3*, *KMT2E*, *SIRPA* and *SYK* had m^6^A modifications in both cell lines (compared to the expression level of *CLUC*), especially *CREBBP* and *FOXO3*, whose modification levels were stronger than the positive control (*GLUC*) (Fig. 5B, C).

In addition to that, m^6^A-IP-qPCR results also showed that compared with other RNAs tested, *CREBBP*, *FOXO3* and *SIRPA* RNAs exhibited significant and consistent changes in their m^6^A levels upon FTO knockdown in both MV4-11 (Fig. 5D and Fig. S5A) and THP-1 cell lines (Fig. 5E and Fig. S5B). These results suggested that these three genes are the potential targets of FTO.

### FTO regulates the chemotherapy resistance of cells through *FOXO3*

**Fig. 6:**
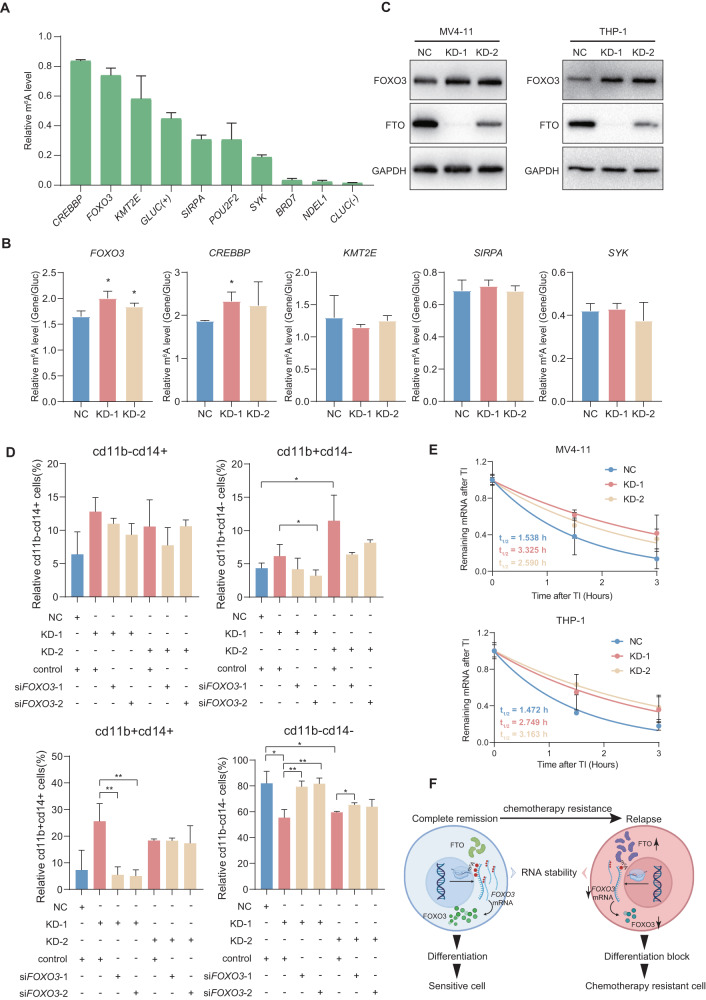
The identification of FTO targets with Ara-C treatment. **A** m^6^A-IP-qPCR results showing the level of m^6^A modification in MV4-11 cells with Ara-C treatment. *GLUC* represents positive control and *CLUC* represents negative control. **B** m^6^A-IP-qPCR results showing the effects of FTO knockdown on the m^6^A levels in MV4-11 cells with Ara-C treatment. **C** Western blot analysis to detect the expressions of FOXO3 proteins before and after knocking down FTO in MV4-11 (left) and THP-1 (right) cell lines. GAPDH was used as a loading control. **D** Flow cytometry to determine the effects of FOXO3 knockdown in rescuing cell differentiation capability of FTO knockdown cells. **E** RNA stability assay to determine the effect of FTO knockdown on the half-lives (*t*_1/2_) of FOXO3 mRNA in MV4-11 (upper panel) and THP-1 (under panel) cells. TI, transcript inhibition. **F** Working model showing the molecular mechanism of FTO in regulating chemotherapy resistant AML cells. Elevated expression of FTO leads to RNA m^6^A hypomethylation of FOXO3, which further affected its RNA stability. Downregulation of FOXO3 results in cell differentiation block, which ultimately leads to chemotherapy resistance and AML relapse. Arrows indicate the sequential changes in gene transcription and expression caused by overexpression of FTO. Representative results from three replicates were shown here. *P*-values were calculated using Two-tailed Student’s *t*-test, and *P* < 0.05 was considered as statistically significant. *P < 0.05, **P < 0.01.

To further identify the targets affecting differentiation, we examined the m^6^A modification of FTO targets under low dose Ara-C treatment. *FOXO3* and *CREBBP* remained highly methylated level (Fig. 6A) and elevated methylation in cells with FTO knocked down (Fig. 6B and Fig. S5C), implying that these two genes may be FTO targets involved in regulating differentiation process.

As we all known, protein is the final form that exerts biological functions, dysregulated of m^6^A methylation leads to the change of protein level [33, 45]. The increase of FOXO3 protein level in FTO knockdown cells implied that served as a target of FTO and assisted in regulating differentiation (Fig. 6C). In order to identify whether FTO regulated the cell differentiation through FOXO3, we found the ratio of CD11b+ or CD14+ cells were partially decreased after knocking down of FOXO3 for further rescue (Fig. 6D and Fig. S5D, S5E), suggesting a role of cell differentiation mediated by FTO in regulating FOXO3 expression level.

To investigate the mechanism, the half-life of *FOXO3* mRNA was increased in FTO knockdown cells compared to negative control cells after treating with actinomycin D (Fig. 6E). However, there was no significant difference of the *FOXO3* mRNA localization (Fig. S5F, S5G), which indicates that the increase of *FOXO3* mRNA stability may lead to the increase of its protein level. In conclusion, FTO-m^6^A-FOXO3 is the main regulatory axis, which affects the chemotherapy resistance of tumor cells by mediating differentiation in the case of abnormal modification of m^6^A.

## Discussion

RNA m^6^A methylation is the most abundant RNA modification in mammals, abnormal expression of m^6^A regulatory factors causes dysregulation of m^6^A modification, which leads to biological dysfunction [46]. It has been proved that dysregulation of epigenetic modifications is a common feature of most human cancers [26]. Currently, the resistance of tumor cells to chemotherapy is still a major obstacle in cancer treatment, the role of m^6^A modification in chemical stimulus response remains to be studied.

In this study, we found that m^6^A modification took parts in “remission-relapse” process through MeRIP-seq, and hypomethylated genes were associated with cell differentiation. We next confirmed that m^6^A demethylase FTO was highly expressed in samples that relapse after chemotherapy, combined with the ability of FTO to regulate cell differentiation [27], we picked FTO to investigate its effect on recurrence. Our results verified that FTO knocked down reduces the resistance of AML cells to chemotherapy at both in vitro and in vivo levels. In terms of mechanism, FOXO3, a factor capable of regulating cell differentiation [47–49], has been proved to be a downstream target of FTO and assist in influencing chemotherapy resistance (Fig. 6F).

Currently, recurrence is still the leading cause of death in AML, which usually related to chemotherapy resistance. Yang et al. pointed out the FTO expression level upregulated in human melanoma, knockdown of FTO sensitized melanoma to anti-PD-1 treatment via adaptive immunity [41]. Besides of that, Lin et al. demonstrated FTO played a critical role in sensitivity of chemo-resistant colorectal cancer cells to 5-fluorouracil, which enhanced tolerance through the activity of autophagy [50]. FTO also has been proved contribute to the chemo-radiotherapy resistance of cervical squamous cell carcinoma [42]. Interestingly, the development of resistance phenotype during target therapy with tyrosine kinase inhibitor (TKI) depends on the m^6^A reduction resulting from FTO overexpression [51]. In consistent with all above findings, here, we demonstrated that FTO took part in sensitivity to chemotherapy in AML, which enhanced resistance via regulating cell differentiation. Similar results were found in both solid and hematologic tumors, so we suggest that m^6^A, especially FTO, regulates chemotherapy resistance and recurrence in tumor. Besides of that, the combination of FTO inhibitors and conventional chemotherapy may be a more effective strategy for cancer therapy.

From previously published studies, FTO plays tumor-promoting roles in various cancers, such as AML [27], glioblastoma [52], breast cancer [53], gastric cancer [54], etc. Li et al. pointed out that the FTO-knockdown decreased undifferentiated NB4 cells (CD11b-/CD14-) by all-trans retinoic acid (ATRA) treatment, otherwise FTO-overexpression increased CD11b-/CD14- cells, indicating that FTO could inhibit ATRA-induced differentiation of APL cells [27]. In consistent with that, we also found an increased tendency of differentiated cells with FTO knocked down after Ara-C treatment, suggesting that cells with high FTO level perform stronger resistance. It is possible that survival of relapsed cells relies more on the FTO.

Based on bioinformatic analysis and experimental validation, we identified FOXO3 as a regulator connecting m^6^A modification with differentiation. FOXO3 was shown a critical role in regulating cell differentiation, overexpression of FOXO3 will induce neural stem cell and follicular helper T cells differentiation [47, 49]. Intriguingly, the mechanism of drug resistance to classical chemotherapeutics are invariably linked to FOXO3-FOXM1 axis [48]. In addition, it has been demonstrated that *FOXO3* was downstream targets of METTL3 in hepato-cellular carcinoma, hypermethylation of *FOXO3* decreased the sorafenib resistance [55]. In this study, we also validated *FOXO3* as a downstream target of FTO in AML. FTO reduction increased m^6^A modification level of *FOXO3*, which leads to increase in protein level. Our results demonstrate that FOXO3 assists in regulating chemotherapy resistance in AML through cell differentiation.

Previous studies indicated a small distribution of m^6^A modification on lncRNAs [56]. There are two mainly opinions being clarified in research into m^6^A and lncRNAs [57]. One is m^6^A modification can regulate the relationship between lncRNA and specific DNA sites [58–60], the other is m^6^A modification on lncRNAs provides binding sites for readers, which then induces RNA binding proteins to regulate the function of lncRNAs [58, 61]. As previously described, we found that there were also m^6^A modification on lncRNAs, and the count increased in AML relapse samples, suggesting they were also involved in the recurrence of AML. We further explored the methylation of lncRNAs associated with tumor progression through bioinformatics analysis and IGV [57], and found there was no significant change between CR and relapse samples, such as *XIST*, *MALAT1* and so on (data not shown). Thus, to fully uncover the roles and mechanisms of lncRNAs in promoting AML relapse, further studies about the relationship between m^6^A and lncRNAs are necessary.

In conclusion, this study demonstrated that m^6^A modification affects disease recurrence in a chemotherapy resistance manner, providing a new perspective for a comprehensive understanding of AML. We uncovered a critical role of m^6^A demethylase FTO in regulating AML chemotherapy resistance in vitro and in vivo. Our study suggests that the FTO-m^6^A-FOXO3 axis acts as a critical regulator in high FTO expression level AML cells, and highlights the functional importance of m^6^A modification machinery in chemotherapy resistance. More importantly, targeting FTO may represents a potential therapeutic strategy to enhance the treatment response in AML patients suffered from chemotherapy resistance.

## Methods and materials

### AML patient samples

The sequential AML samples were obtained at the stage of CR and relapse with informed consent at the Hematology Department of Peking University People’s Hospital, and were approved by the local ethics review board (Reference number: 2023PHB039-001). The leukemic samples were stored in liquid nitrogen until used. Total RNA was extracted through TriReagent (T9424, Sigma, Missouri, USA) following the manufacturer’s protocol.

### Cell culture

The human AML cell lines MV4-11, THP-1, U937, Molm-13, NB4 and HL60 were maintained in the laboratory. All cell lines have been authenticated and tested as free of mycoplasma contamination. All cell lines were grown in RPMI medium 1640 (C11875500BT, Gibco, Grand Island, USA) containing 10% FBS (Gibco) and 1% penicillin-streptomycin (10378-016, Gibco).

The FTO-knockdown MV4-11 and THP-1 cell lines were established by using lentivirus system. Cells were transfected with lentivirus particles and followed by 5 μg/ml puromycin selection. The virus was purchased from Vigene Biosciences (Shandong, China) and shRNA sequence employed in this experiment is listed in Key Resources Table.

### Xenograft mice model of AML

Thirty male NOD.Cg-Prkdc^scid^ Il2rg^tm1Vst^/Vst (NPG) mice (6 weeks old) were divided into three groups and implanted with 4 × 10^6^ cells of control (MV4-11 NC) or FTO-knockdown (MV4-11 KD-1 or KD-2) by tail vein injection, respectively. Tumor growth was monitored every week through flow cytometry. Each group of mice were further randomized into two groups and treated with cytarabine (Ara-C) (S1648, Selleck, Texas, USA) or PBS (100 mg/kg, once every two days, three times in all) through tail vein injection when the percentage of GFP+ cells in peripheral blood reached ~5%. The mice were sacrificed and detected the proportion of GFP+ cells in bone marrow when chemotherapy finished. The tumor infiltration in spleen was identified through H&E staining. All the animal experiment were performed according to the guidelines of Animal Care and Use Committee. The Institutional Review Board approval number is 2020PHE006.

### Western blot analysis

Cellular proteins were extracted with RIPA lysis buffer containing protease inhibitor cocktail (#04693159001, Roche, Basel, Switzerland) and phosphatase inhibitor cocktail (#04906837001, Roche). About 2 × 10^5^ cells per lane was applied to SDS-PAGE and immunoblotting analysis. Detailed information of the primary and secondary antibodies used in this study is shown in Key Resources Table. The quantifications of Western blot were performed through ImageJ.

### Cell viability assay

Cells were seeded in density of 2 × 10^4^ per well into 96-well plate and incubate with gradient concentrations of Ara-C (#S1648, Selleck) or doxorubicin (DOX) (#S1208, Selleck) for another 48 h. Cell viability was determined through Cell Counting Kit-8 (CCK8) (#CK04, DOJINDO, Kumamoto Prefecture, Japan) following the manufacturer’s protocol. The absorbance was detected at 450 nm.

### Flow cytometry analysis

For in vitro cell differentiation assay, FTO knockdown and control cells were incubated with 250 ng/μl Ara-C (#S1648, Selleck) for 48 h, cells were harvested and washed with chilled PBS and stained with PE-labeled anti-CD11b (#301306, BioLegend, California, USA) and APC-labeled anti-CD14 (#367118, Biolegend) for flow cytometry analysis. The fluorescence intensity was detected using CANTO PLUS (BD Biosciences, New Jersey, USA) and analyzed with the FlowJo.

### Construction of Ara-C-resistant cell line

MV4-11 cells were used for constructing drug-resistant cell line. Cells were treated with 1/10 of the wildtype cell IC50 at the initial for 24 h and then transferred to drug-free medium. The concentration of Ara-C was increased and the incubation continued in the same way until the cells were able to grow stably at the high dose of Ara-C.

### Subcellular fractionation analysis of RNA

FTO knockdown and control cells were subjected to subcellular fraction using NE-PER Nuclear and Cytoplasmic Extraction Reagents (#78833, Thermo Fisher) according to the manufacturer’s manual. 200 μl of the cytoplasm extract and 100 μl of the nucleus extract were used for total RNA extraction, reverse transcription and qPCR. *ND4* was used as separation marker.

### RNA stability assay

For RNA stability assay, MV4-11 and THP-1 cells (FTO knockdown and control) were incubated with 5 μg/ml Actinomycin D (#A9415, Sigma). Cells were harvested at 0 h, 1 h, 2 h, and 3 h post treatment and subjected to RT-qPCR for quantitative analysis of target genes. *18S* was used as negative control. The degradation rate of RNA was calculated using the following equation:

$${{{\mathrm{Nt}}}}/{{{\mathrm{N}}}}0 = {{e}}^{ - {{kt}}}$$

where *t* is the duration of transcription inhibition, *k* is the degradation rate, Nt and N0 are the RNA quantities at time *t* and time 0. The half-life of RNA (*T*_1/2_) can be calculated from the degradation rate as follows:

$${{T}}_{1/2} = {{{\mathrm{ln}}}}2/{{k}}$$

### m^6^A-seq and m^6^A-IP-qPCR

For total RNA m^6^A-seq, it was performed as previously described with miner revision [62]. In brief, total RNA was treated with DNase I (#04716728001, Roche) first to remove genomic DNA. Next, total RNA was fragmented to 100–150 nt by using RNA Fragmentation Reagents (#AM8740, Thermo Fisher, Waltham, USA). At least 10 μg fragmented total RNA was incubated with 5 μg anti-N6-methyladenosine antibody (#202003, Synaptic Systems, Gottingen, Germany) at 4 °C for 6 h, followed by incubation with DynabeadsTM Protein A (#10002D, Thermo Fisher) for another 3 h. Immunoprecipitated RNA was eluted with RLT buffer and recovered through RNeasy Mini Kit (#74106, Qiagen, Germantown, USA). cDNA libraries were constructed from immunoprecipitated RNAs and input RNAs, respectively, by using SMARTer Stranded Total RNA-Seq Kit v2 (#634413, Takara, Kyoto, Japan), and then subjected to next-generation sequencing on NovaSeq platform.

For gene-specific m^6^A-IP-qPCR, 20 μg of fragmented total RNA (300–500 nt) added 0.1 fmol of positive spike-in control was used for experiment. The m^6^A-IP was performed by using the N6-methyladenosine Enrichment kit (E1610S, New England Biolabs, Ipswich, USA). Reverse transcription was performed by using ReverTra Ace qPCR RT Master Mix (#FSQ-301, TOYOBO, Osaka, Japan). RT-qPCR was performed by using PowerUP SYBR Green Master Mix (A25742, Invitrogen, California, USA). GAPDH was used as internal control. The sequences of primers used in this study are listed in Key Resources Table.

### Processing of high-throughput sequencing data

Trimmomatic software were used to remove adapter and low-quality reads of raw sequencing data [63]. Quality distribution plot and base content distribution were generated by FASTQC [Available: http://www.bioinformatics.babraham.ac.uk/projects/fastqc/]. Clean reads were aligned to the human genome (build 38) using STAR [62], which were used for subsequent analysis.

### m^6^A-seq data analysis

The m^6^A peak was identified through MetPeak R package [64] in each m^6^A-IP sample with the corresponding input sample as control. The identification of m^6^A peaks followed MetPeak; PEAK_CUTOFF_*P* value = 0.05, FOLD_ENRICHMENT = 1 were considered as high-confidence peaks and used for further analysis. The density plot of peaks in 3'UTR, CDs and 5'UTR was completed with Guitar [65].

### Differential methylation analysis

To determine differential m^6^A methylation modifications between CR and relapse samples, the MeTDiff R package effectively modeled the reads of methylation data and predicted the differential methylation sites by using the maximum likelihood ratio test. High-confidence differential m^6^A peaks were determined with the criteria of “*P*-value < 0.05, | log2 (fold-change) | > 1”.

### GO analysis

Gene ontology (GO) analysis was performed to facilitate elucidating the biological implications of unique genes in the significant or representative profiles of the gene in the experiment [66]. We downloaded the GO annotations from Ensembl (http://asia.ensembl.org/index.html), and the Gene Ontology (http://www.geneontology.org/). ClusterProfiler was applied to identify the significant GO categories and FDR was used to correct the *P*-values.

### Quantification and statistical analysis

Statistical analyses were performed using Graphpad Prism 8.0 software. *P*-values were calculated using Two-tailed Student’s *t*-test or Wilcoxon test, and *P* < 0.05 was considered as statistically significant. Data are shown as mean ± SD. At least three biological replicates were included in each experiment.

## Supplementary information

Supplementary information

Supplementary table S1

Supplementary table S2

Supplementary table S3

Supplementary table S4

Supplementary table S5

Full and uncropped western blots

Key resources table

## Data Availability

The datasets generated during and/or analyzed during the current study are available from the corresponding author on reasonable request. The raw sequence data reported in this paper have been deposited in the Genome Sequence Archive [67] in National Genomics Data Center [68], China National Center for Bioinformation/Beijing Institute of Genomics, Chinese Academy of Sciences (GSA-Human: HRA003825) that are publicly accessible at https://ngdc.cncb.ac.cn/gsa-human.
