# Supplementary material for: The role of m6A demethylase FTO in chemotherapy resistance mediating acute myeloid leukemia relapse
Source: Cell Death Discov. 2023 Jul 5;9:225. doi: 10.1038/s41420-023-01505-y (PMC10319875; doi:10.1038/s41420-023-01505-y)
Supplement: Supplementary file 1 — Supplementary information [file 41420_2023_1505_MOESM1_ESM.pdf]

Supplementary Information

The role of m6A demethylase FTO in chemotherapy resistance mediating acute myeloid leukemia relapse

Zhi-Wei Zhang[1], Xiao-Su Zhao[1], Huidong Guo[1], Xiao-Jun Huang[1, 2*]

* **Corresponding author**

Huang Xiao-Jun, (xjhrm@medmail.com.cn) +86-010-88326006

Peking University People's Hospital & Peking University Institute of Hematology, National Clinical Research Center for Hematologic Disease, Beijing Key Laboratory of Hematopoietic Stem Cell Transplantation, Peking University, Beijing, 100044, China.

Peking-Tsinghua Center for Life Sciences, School of Life Sciences, Peking University, Beijing, 100044, China.

Supplementary Information includes:

Supplementary Figure S1-S5

Supplementary Table S1-S5 (see additional files)

Key Resources Table (see additional files)

Full and uncropped western blots (see additional files)

Figure S1

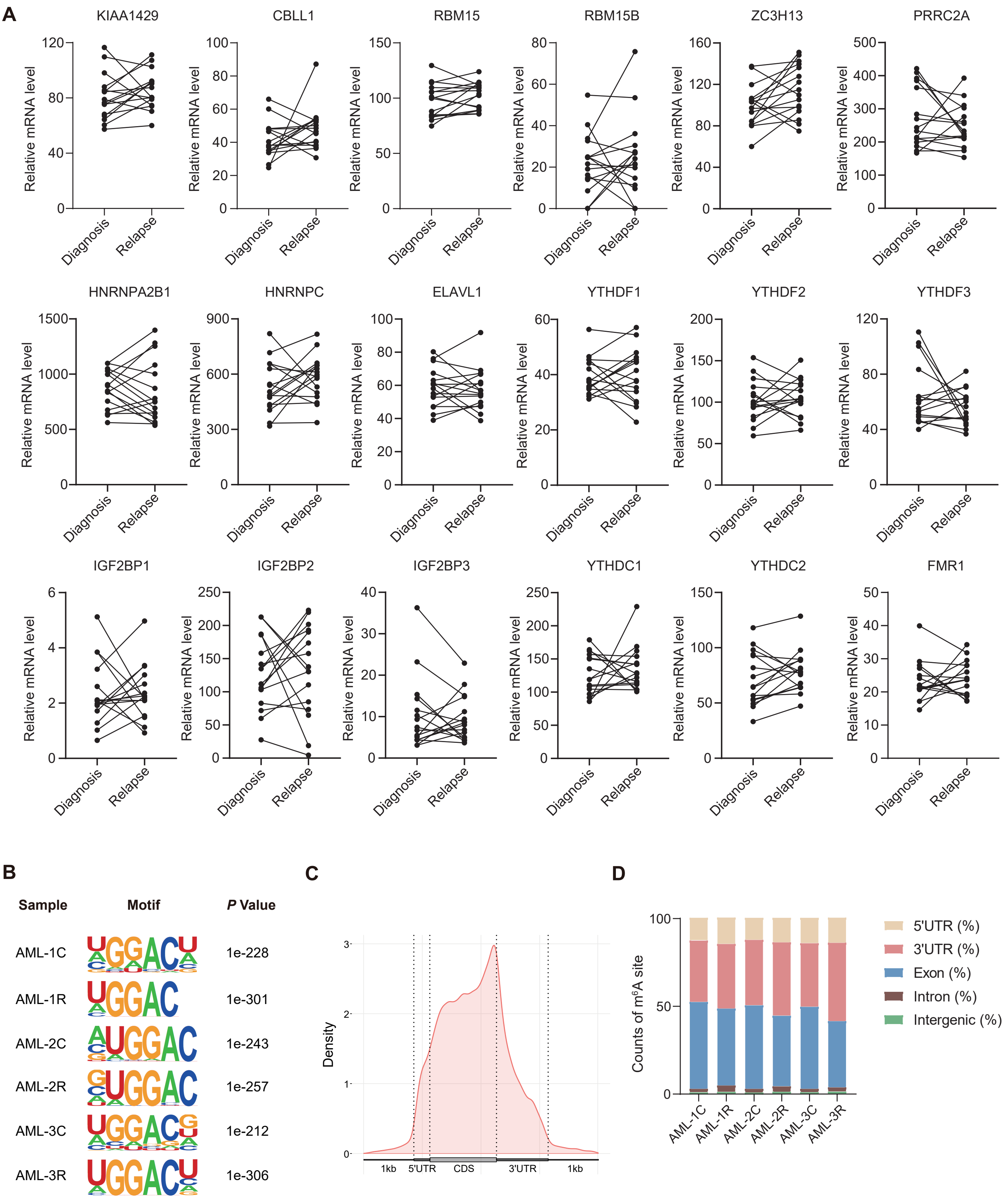
A
KIAA1429
CBLL1
RBM15
RBM15B
ZC3H13
PRRC2A
HNRNPA2B1
HNRNPC
ELAVL1
YTHDF1
YTHDF2
YTHDF3
IGF2BP1
IGF2BP2
IGF2BP3
YTHDC1
YTHDC2
FMR1
Relative mRNA level
Diagnosis
Relapse
B
Sample
Motif
P Value
AML-1C 1e-228
AML-1R 1e-301
AML-2C 1e-243
AML-2R 1e-257
AML-3C 1e-212
AML-3R 1e-306
C
Density
1kb
5'UTR
CDS
3'UTR
1kb
D
Counts of m6A site
AML-1C
AML-1R
AML-2C
AML-2R
AML-3C
AML-3R
5'UTR (%)
3'UTR (%)
Exon (%)
Intron (%)
Intergenic (%)

Figure S1. Transcriptome-wide RNA m^{6}A methylation analysis in complete remission and relapse samples. Related to Figure 1. A. Comparison of RNA expression level of m^{6}A regulators between sequential diagnosis (n = 16) and relapse samples (n = 16) from a public dataset. *P* values were calculated using Wilcoxon test, and $P < 0.05$ was considered as statistically significant. All genes in the graph were not significant. **B.** Top consensus motif of m^{6}A peaks identified in three pairs of sequential samples. C: complete remission; R: relapse. **C.** Metagene profiles showing the distribution of m^{6}A peaks across the mRNA transcripts, representative figure of 6 samples was shown here. **D.** The statistics of m^{6}A peaks enriched in different regions on mRNA transcripts.

Figure S2

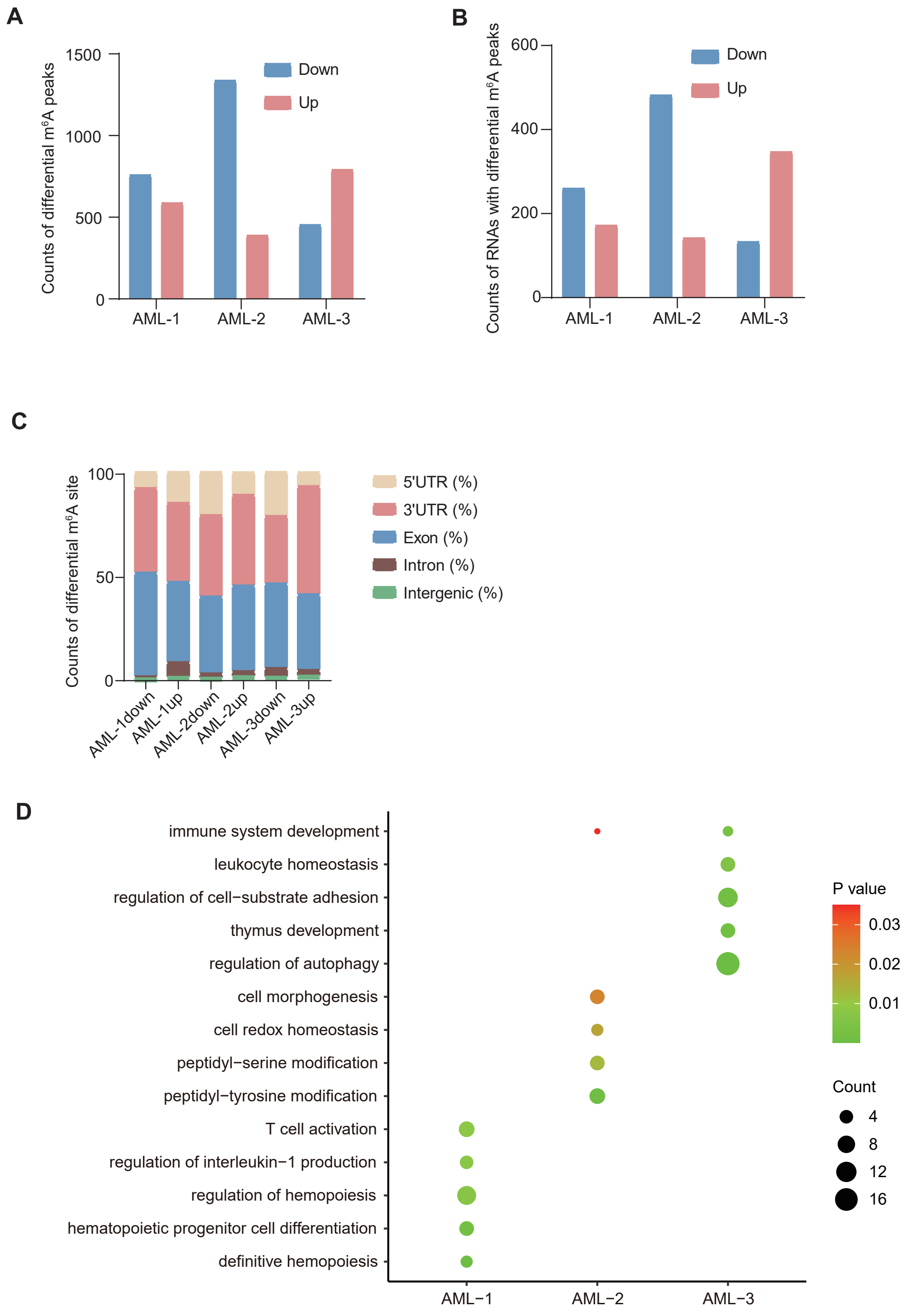
A
Counts of differential m6A peaks
1500
1000
500
0
Down
Up
AML-1
AML-2
AML-3
B
Counts of RNAs with differential m6A peaks
600
400
200
0
Down
Up
AML-1
AML-2
AML-3
C
Counts of differential m6A site
100
50
0
5'UTR (%)
3'UTR (%)
Exon (%)
Intron (%)
Intergenic (%)
AML-1down
AML-1up
AML-2down
AML-2up
AML-3down
AML-3up
D
immune system development
leukocyte homeostasis
regulation of cell−substrate adhesion
thymus development
regulation of autophagy
cell morphogenesis
cell redox homeostasis
peptidyl−serine modification
peptidyl−tyrosine modification
T cell activation
regulation of interleukin−1 production
regulation of hemopoiesis
hematopoietic progenitor cell differentiation
definitive hemopoiesis
AML−1
AML−2
AML−3
P value
0.03
0.02
0.01
Count
4
8
12
16

Figure S2. Analysis of transcriptome-wide differential RNA m^{6}A methylation in three pairs of AML sequential samples. Related to Figure 2. A. Bar graph showing the counts of up-regulated and down-regulated m^{6}A peaks in AML relapse samples. **B.** The statistics of the counts of RNAs with up-regulated and down-regulated m^{6}A peaks identified in three pairs of sequential samples. **C.** Bar graph showing the differential m^{6}A peaks enriched in different regions on mRNA transcripts. **D.** GO analysis of the hyper-methylated RNAs in relapse samples.

Figure S3

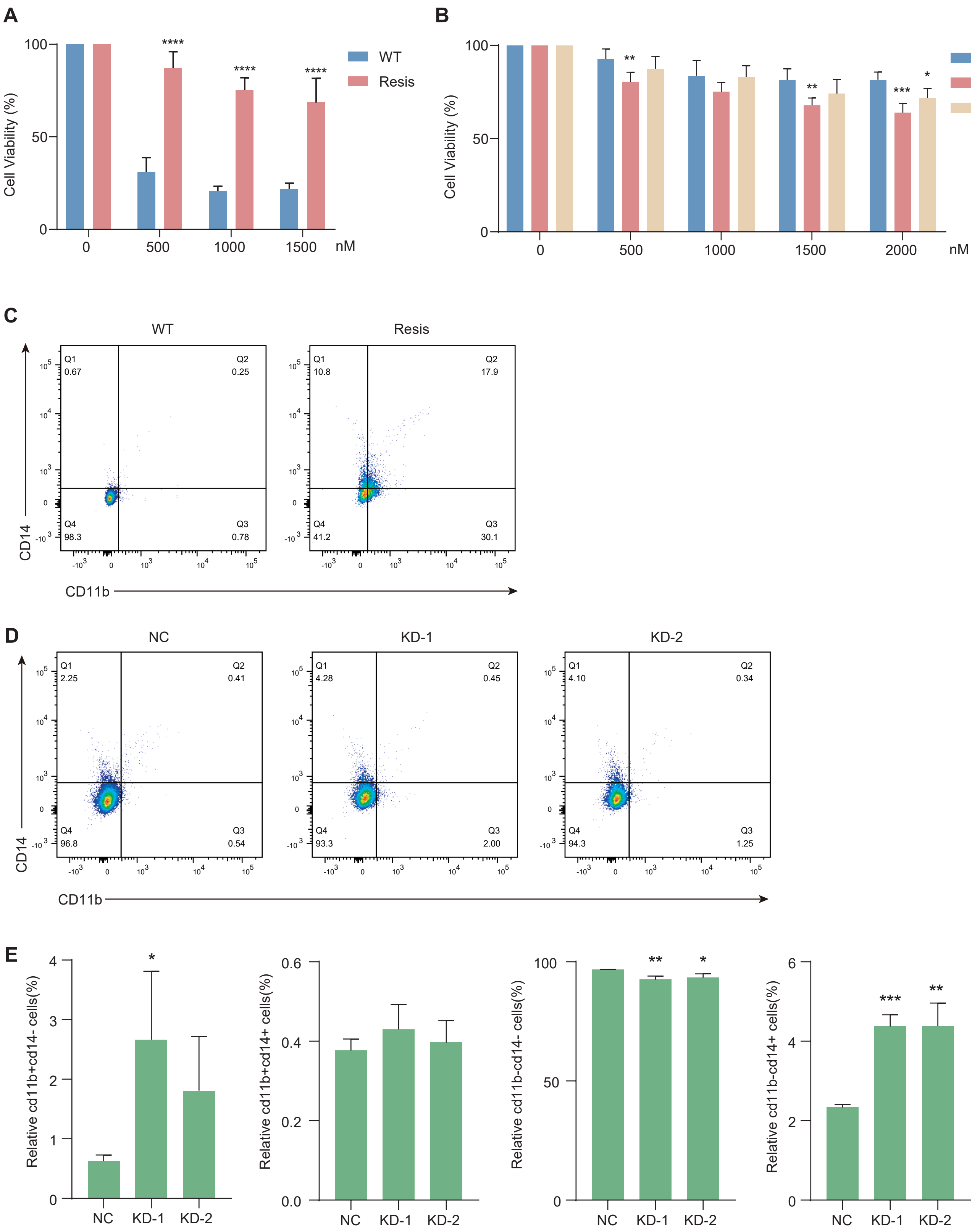
A
Cell Viability (%)
100
50
0
0
500
1000
1500
nM
****
WT
Resis
B
Cell Viability (%)
100
50
0
0
500
1000
1500
2000
nM
**
***
*
NC
KD-1
KD-2
C
WT
Resis
Q1
0.67
Q2
0.25
Q4
98.3
Q3
0.78
Q1
10.8
Q2
17.9
Q4
41.2
Q3
30.1
CD14
CD11b
D
NC
KD-1
KD-2
Q1
2.25
Q2
0.41
Q4
96.8
Q3
0.54
Q1
4.28
Q2
0.45
Q4
93.3
Q3
2.00
Q1
4.10
Q2
0.34
Q4
94.3
Q3
1.25
CD14
CD11b
E
Relative cd11b+cd14- cells(%)
Relative cd11b+cd14+ cells(%)
Relative cd11b-cd14- cells(%)
Relative cd11b-cd14+ cells(%)
NC
KD-1
KD-2

Figure S3. Effects of FTO knockdown on cell viability and differentiation in drug-resistant cell. Related to Figure 3. A. Cell viability assay showing the difference between wildtype (WT) and Ara-C resistant (Resis) MV4-11 cells. **B.** Cell viability assay showing the effects of FTO on chemotherapy resistance with Ara-C treatment in Resis cells. **C.** Flow cytometry detecting the expression pattern of CD11b and CD14 in WT and Resis cells. **D, E.** Flow cytometry showing the regulation of FTO to cell differentiation in Resis cells (**D**). The statistical analysis was shown in (**E**). Representative results from three replicates were shown here. P values were calculated using Two-tailed Student's t-test, and $P < 0.05$ was considered as statistically significant. $^{*}P < 0.05$, $^{**}P < 0.01$, $^{***}P < 0.001$.

Figure S4

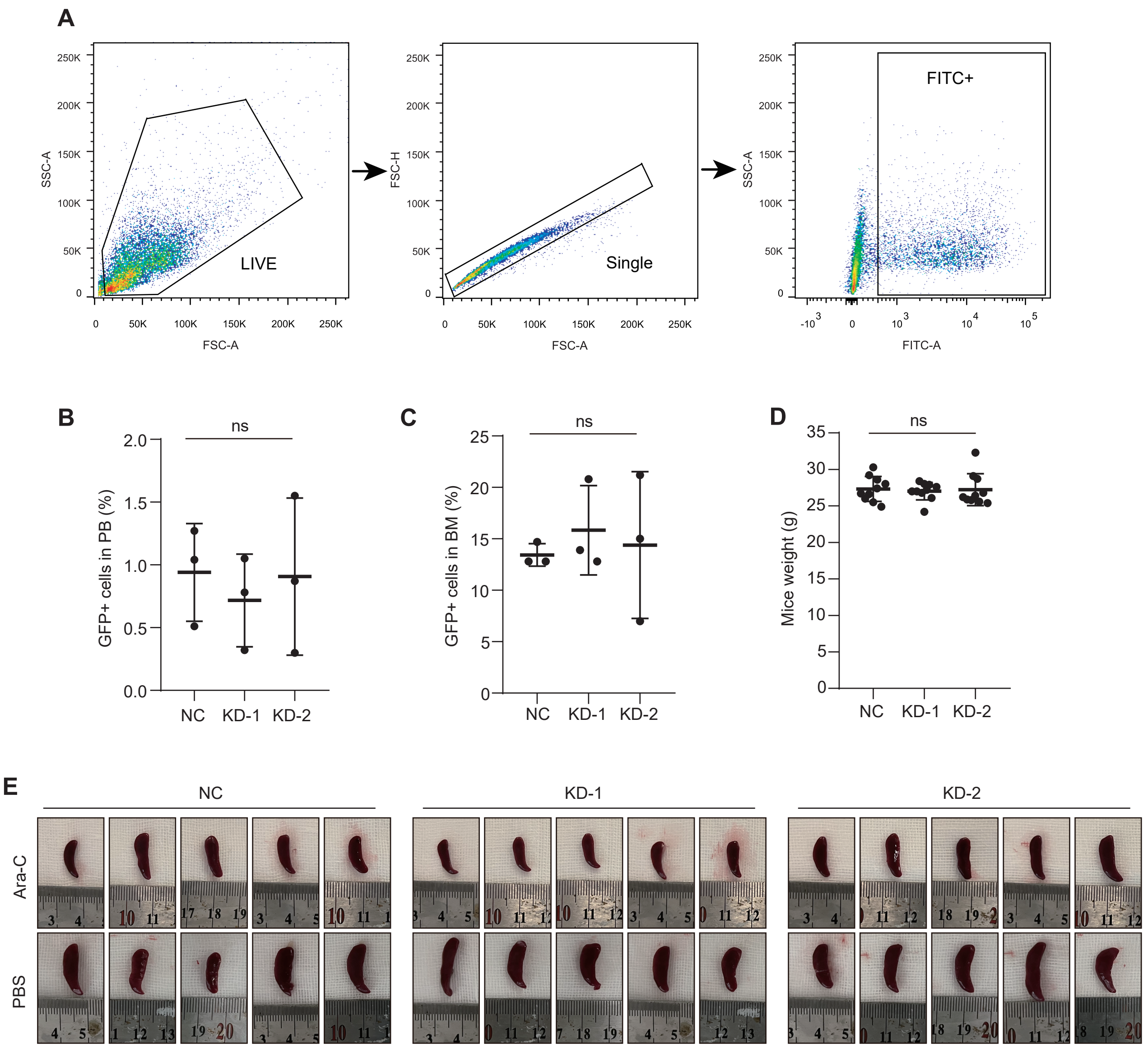
A
LIVE
Single
FITC+
SSC-A
FSC-A
FSC-H
FITC-A
B
GFP+ cells in PB (%)
ns
NC
KD-1
KD-2
C
GFP+ cells in BM (%)
D
Mice weight (g)
E
Ara-C
PBS

Figure S4. Effects of FTO knockdown on chemotherapy resistance in vivo. Related to Figure 4. A. The strategy to determine GFP+ cells in xenograft mice through flow cytometry. **B, C.** Flow cytometry to detect the percentage of GFP+ cells in peripheral blood (**B**) and bone marrow (**C**) separately. **D.** The mice weight detection before sampling. *P* values were calculated using Two-tailed Student's t-test, ns: not significant. **E.** The comparison of spleen size between Ara-C treatment and control groups.

Figure S5

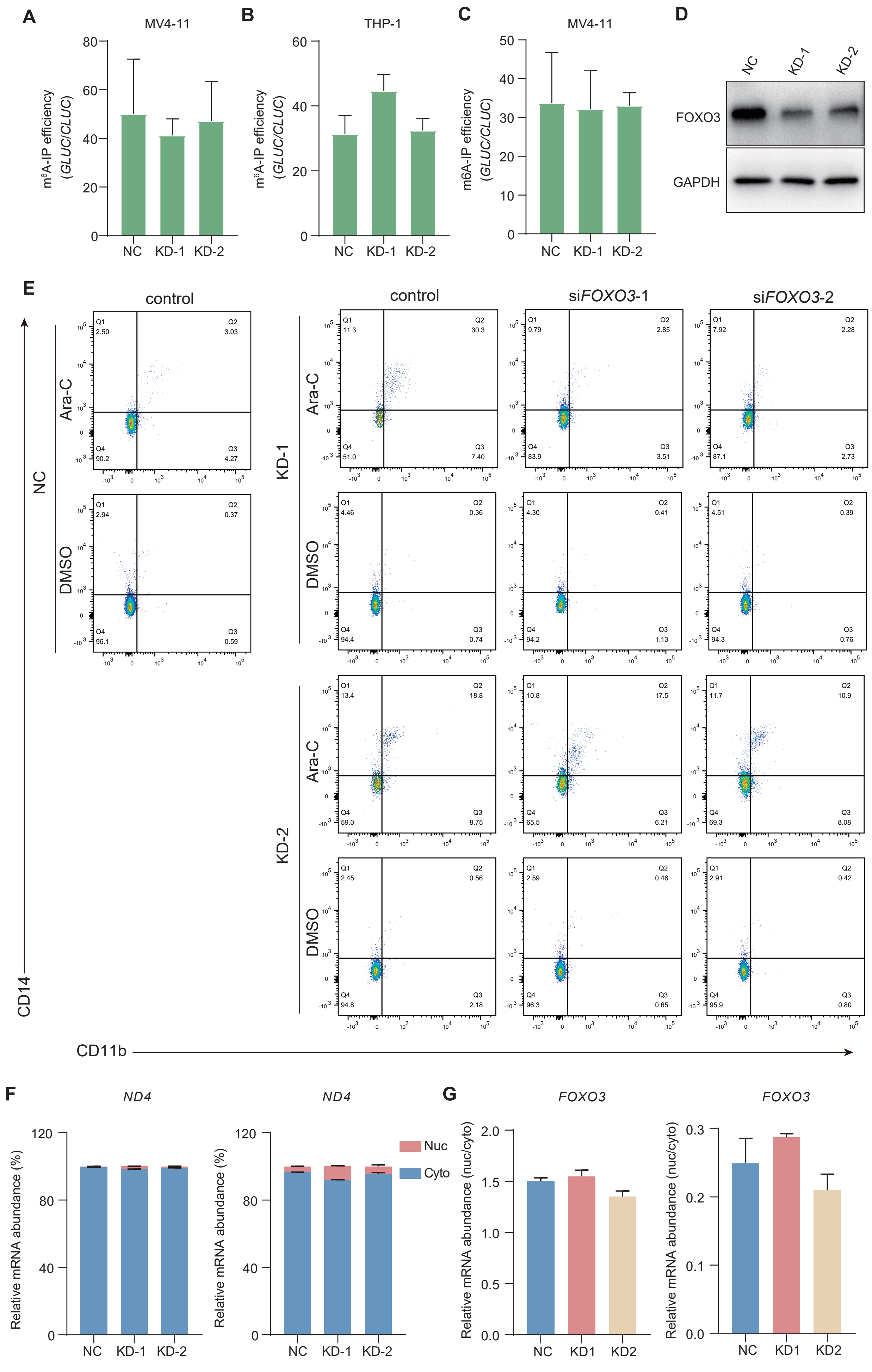

A
MV4-11
m6A-IP efficiency (GLUC/CLUC)
NC KD-1 KD-2
B
THP-1
C
m6A-IP efficiency (GLUC/CLUC)
D
NC KD-1 KD-2
FOXO3
GAPDH
E
control
siFOXO3-1
siFOXO3-2
NC
KD-1
KD-2
Ara-C
DMSO
CD14
CD11b
F
ND4
Relative mRNA abundance (%)
Nuc
Cyto
G
FOXO3
Relative mRNA abundance (nuc/cyto)
NC KD1 KD2

Figure S5. The effects of FTO knockdown on the m⁶A levels and RNA fate. Related to Figures 5, 6. A, B The comparison of m^6A-IP efficiency between NC (negative control) and FTO knock down cells of MV4-11 (**A**) and THP-1 (**B**) cells. **C.** The detection of m^6A-IP efficiency between NC and FTO knock down cells in MV4-11 cells with Ara-C treatment. The IP efficiency of each group is consistent. **D.** Western blot analysis confirming the effects of FOXO3 knockdown, GAPDH was used as loading control. **E.** Flow cytometry to determine the effects of FOXO3 knockdown in rescuing cell differentiation capability of FTO knockdown cells. The statistical analysis was shown in (**Fig. 6D**). **F.** Relative RNA abundance of cytoplasmic marker *ND4*. **G.** Subcellular localization of *FOXO3* mRNA was shown by the ratio of RNA abundance between the cytoplasmic and nuclear fractions in MV4-11 (left panel) and THP-1 (right panel) cells. Representative data from three independent experiments were shown here. P values were calculated using Two-tailed Student's t-test, and $P < 0.05$ was considered as statistically significant.
