# Supplementary material for: The role of m6A demethylase FTO in chemotherapy resistance mediating acute myeloid leukemia relapse
Source: Cell Death Discov. 2023 Jul 5;9:225. doi: 10.1038/s41420-023-01505-y (PMC10319875; doi:10.1038/s41420-023-01505-y)

Full and uncropped western blots

The role of m^6A demethylase FTO in chemotherapy resistance mediating acute myeloid leukemia relapse

Zhi-Wei Zhang[1], Xiao-Su Zhao[1], Huidong Guo[1], Xiao-Jun Huang[1, 2*]

* **Corresponding author**

Huang Xiao-Jun, (xjhrm@medmail.com.cn) +86-010-88326006

Peking University People's Hospital & Peking University Institute of Hematology, National Clinical Research Center for Hematologic Disease, Beijing Key Laboratory of Hematopoietic Stem Cell Transplantation, Peking University, Beijing, 100044, China.

Peking-Tsinghua Center for Life Sciences, School of Life Sciences, Peking University, Beijing, 100044, China.

- **Figure 3B**

- FTO

Original

FTO

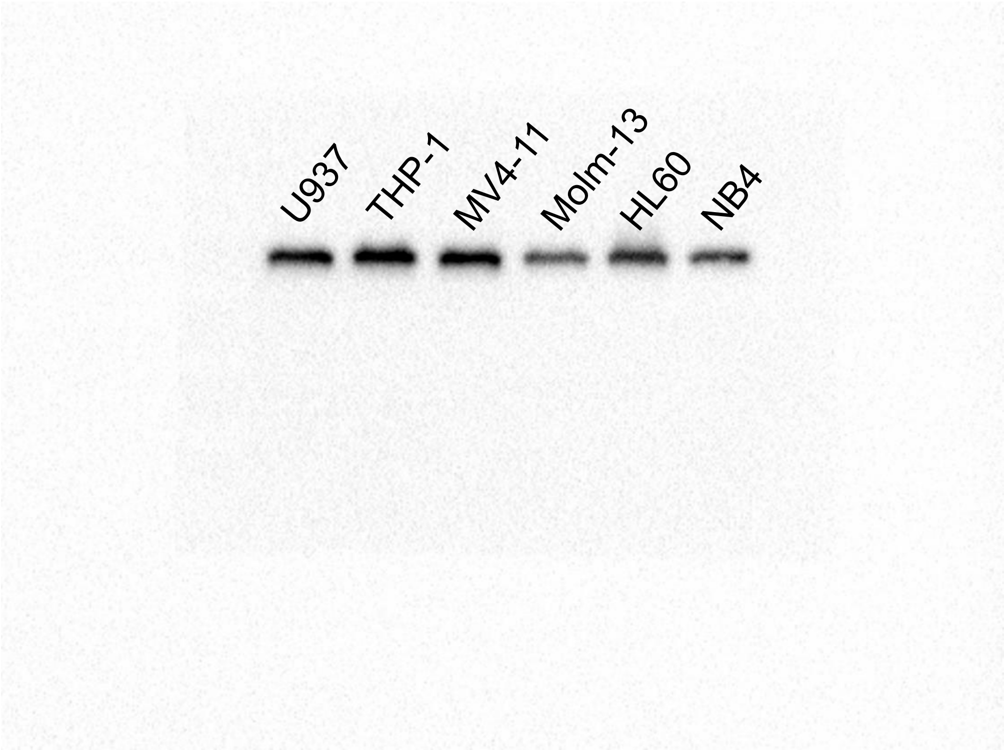

Merge

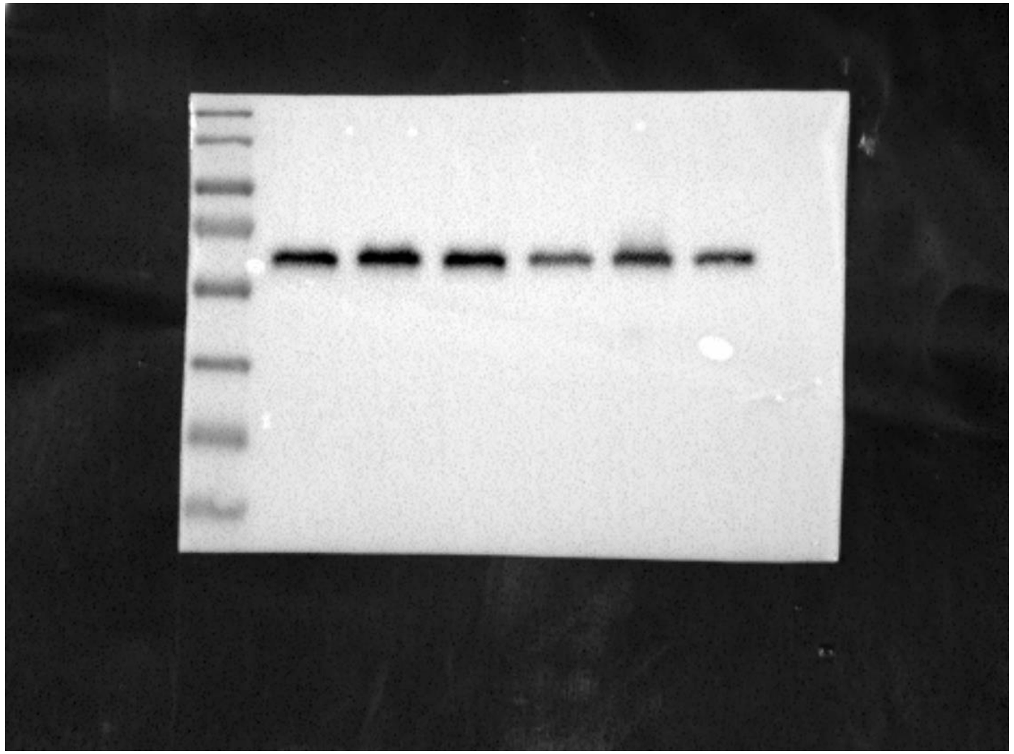

➢ **Figure 3B**

- GAPDH

Original

GAPDH

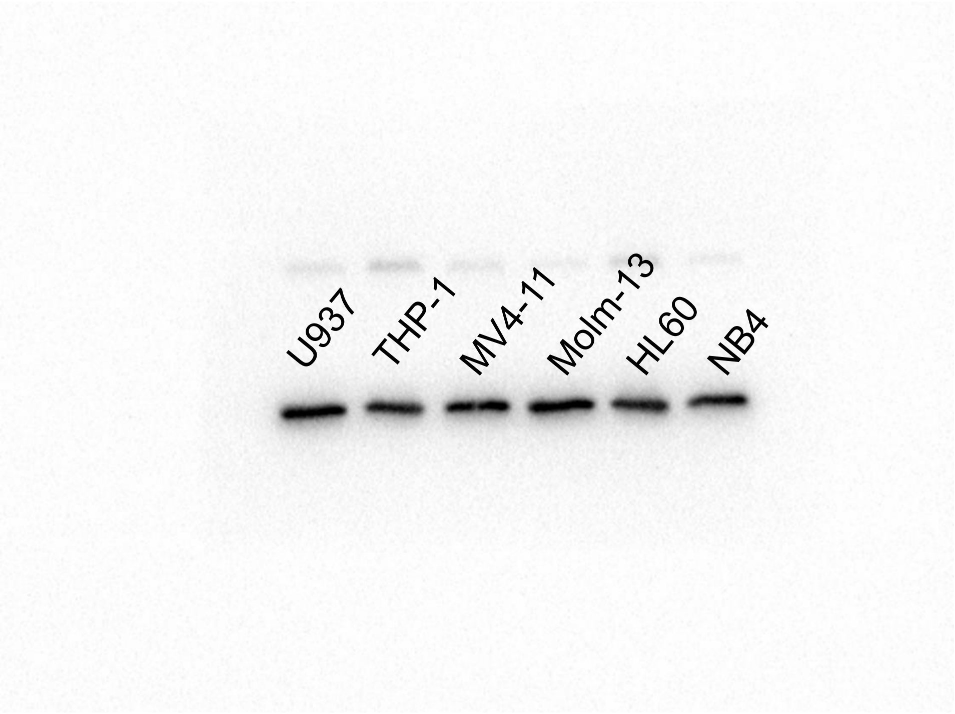

Merge

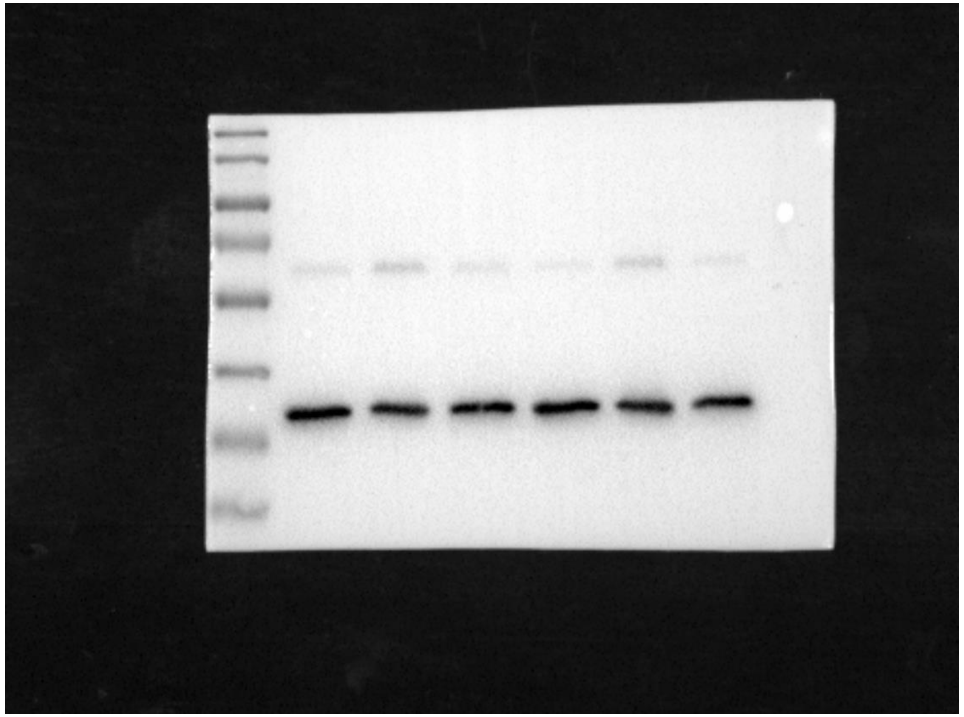

➢ **Figure 3C**

- MV4-11 FTO

Original

FTO

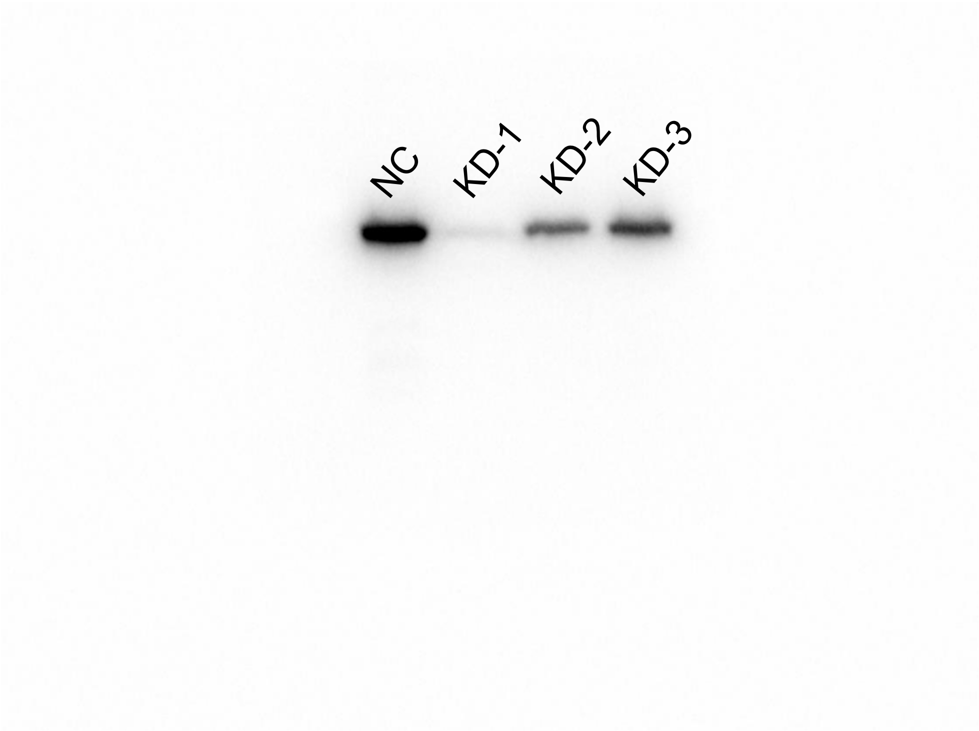

Merge

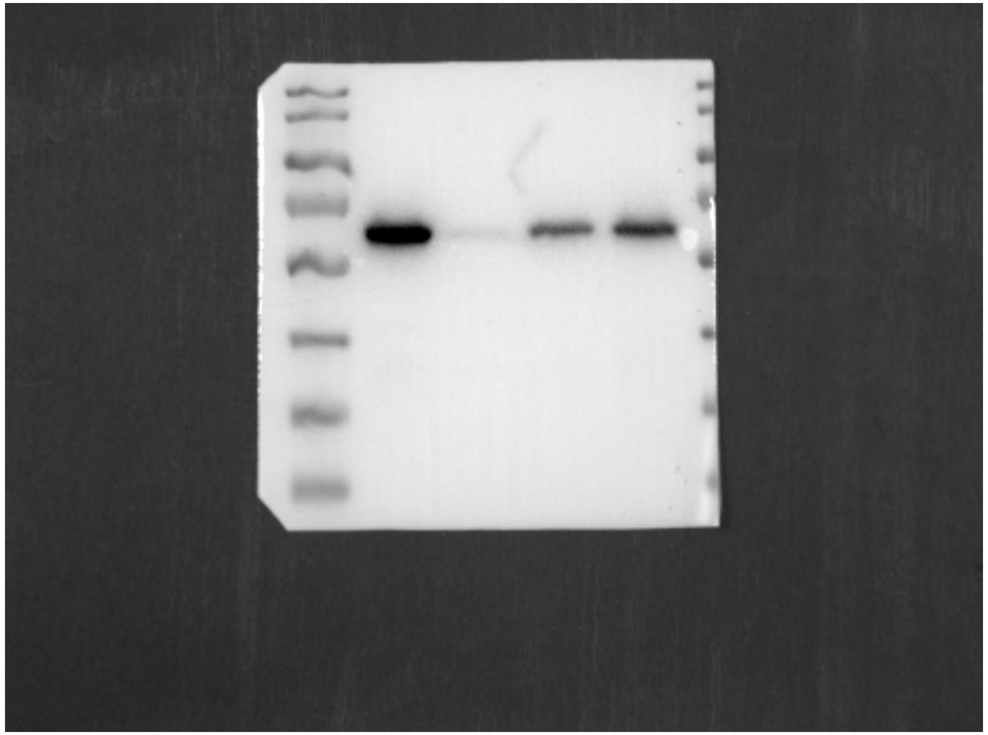

➢ **Figure 3C**

- MV4-11 GAPDH

Original

Merge

GAPDH

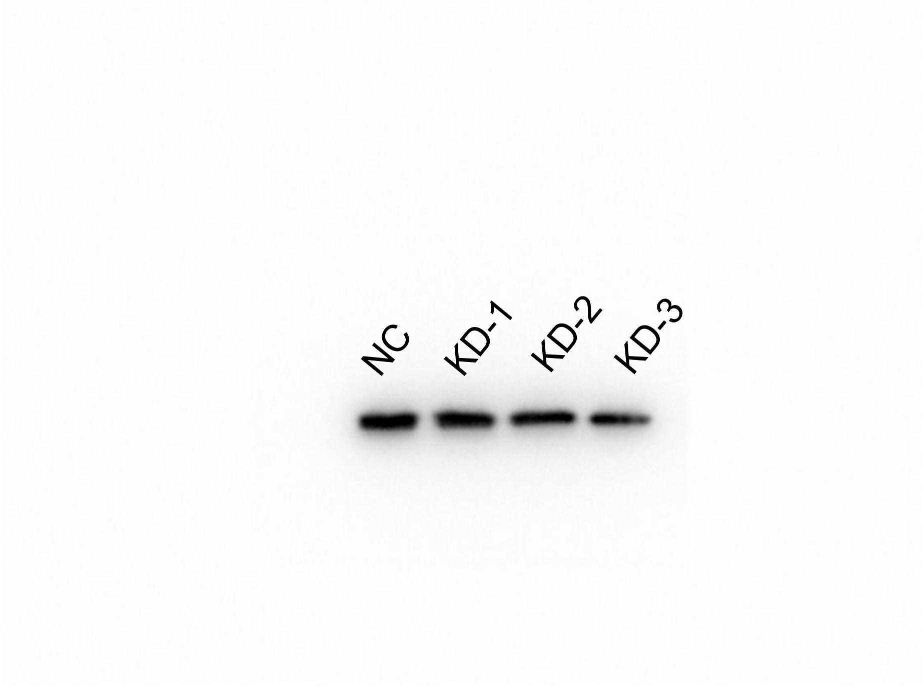

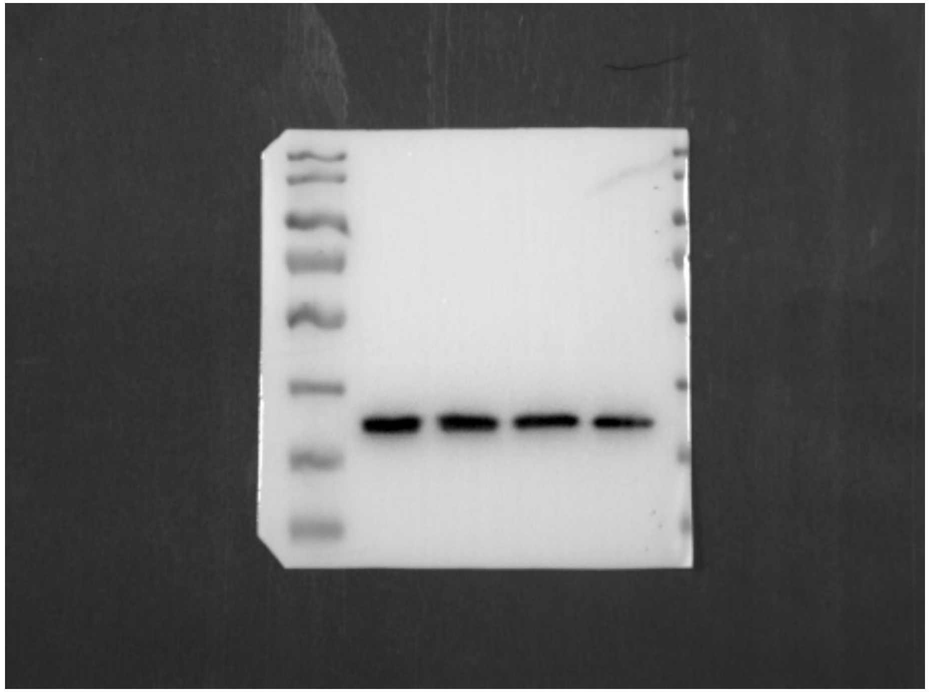

➢ **Figure 3C**

- THP-1 FTO

Original

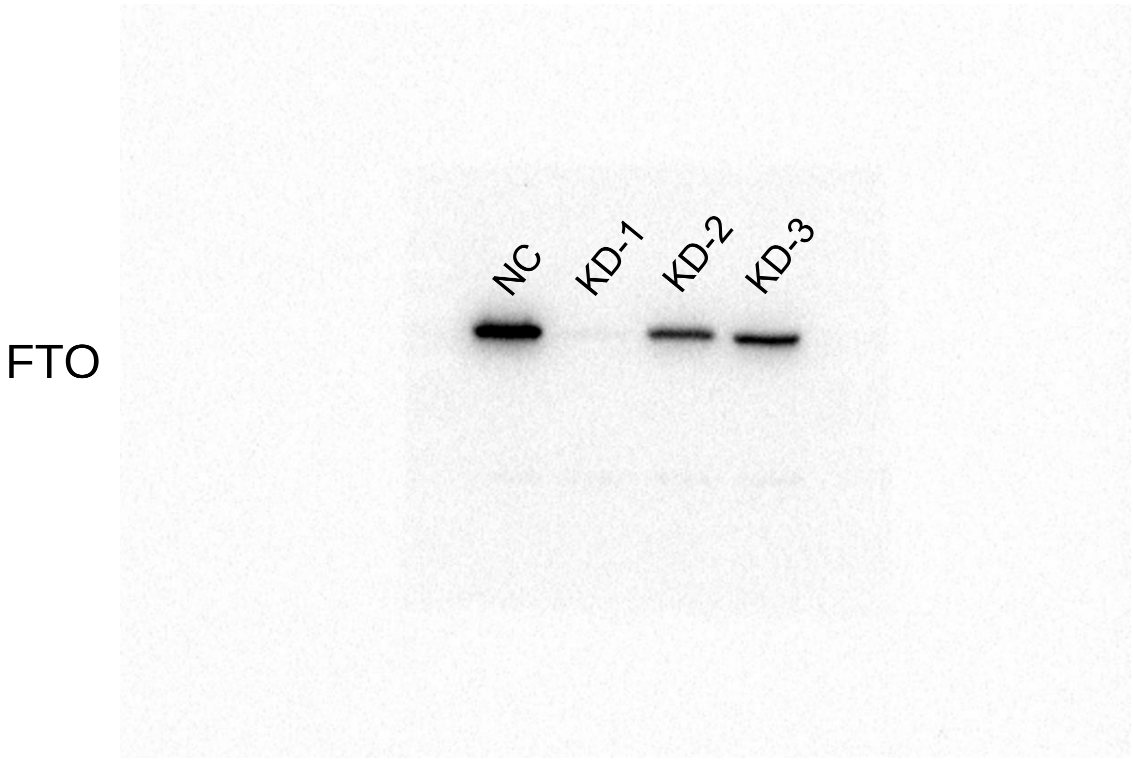

Merge

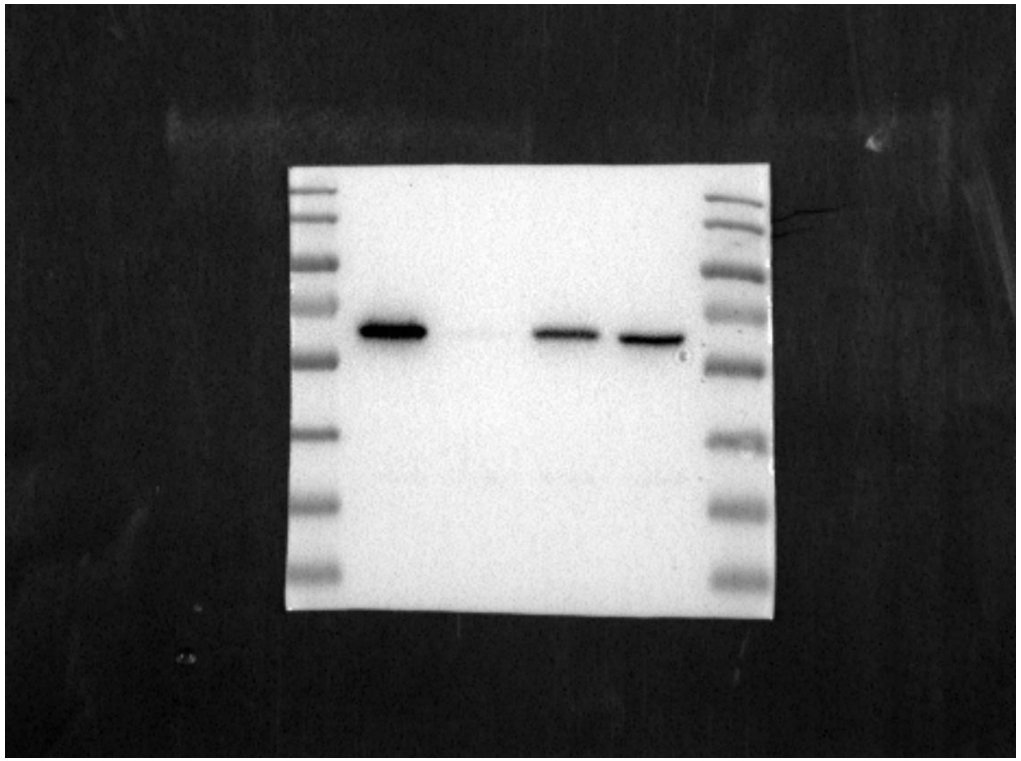

➢ **Figure 3C**

- THP-1 GAPDH

Original

Merge

GAPDH

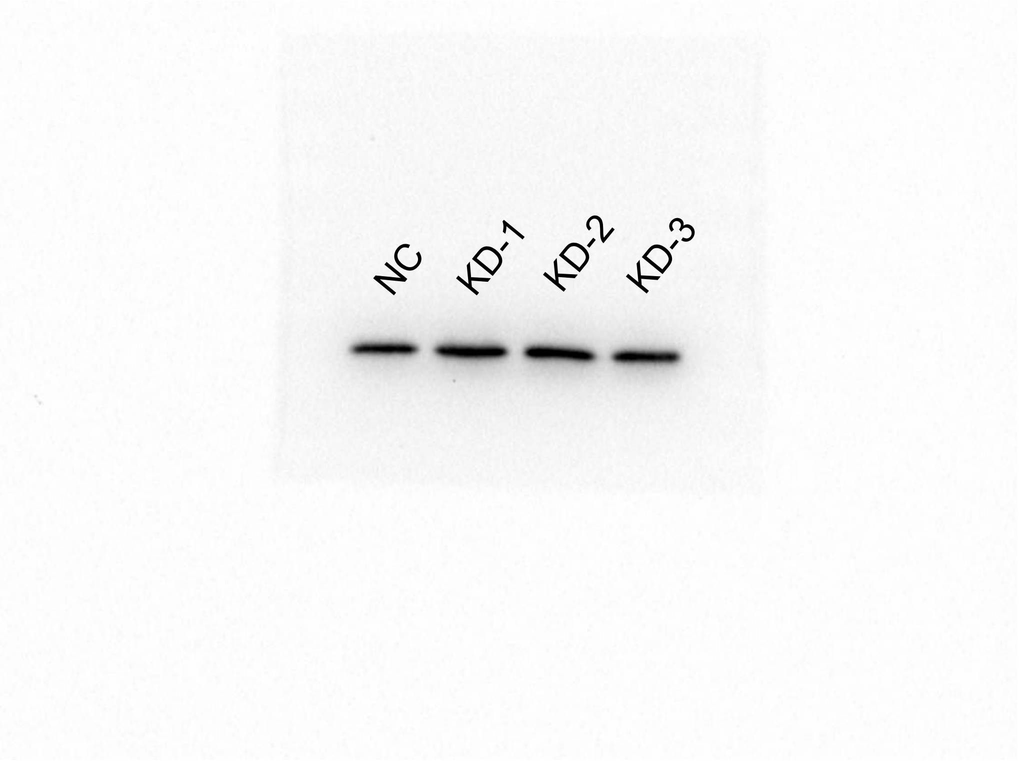

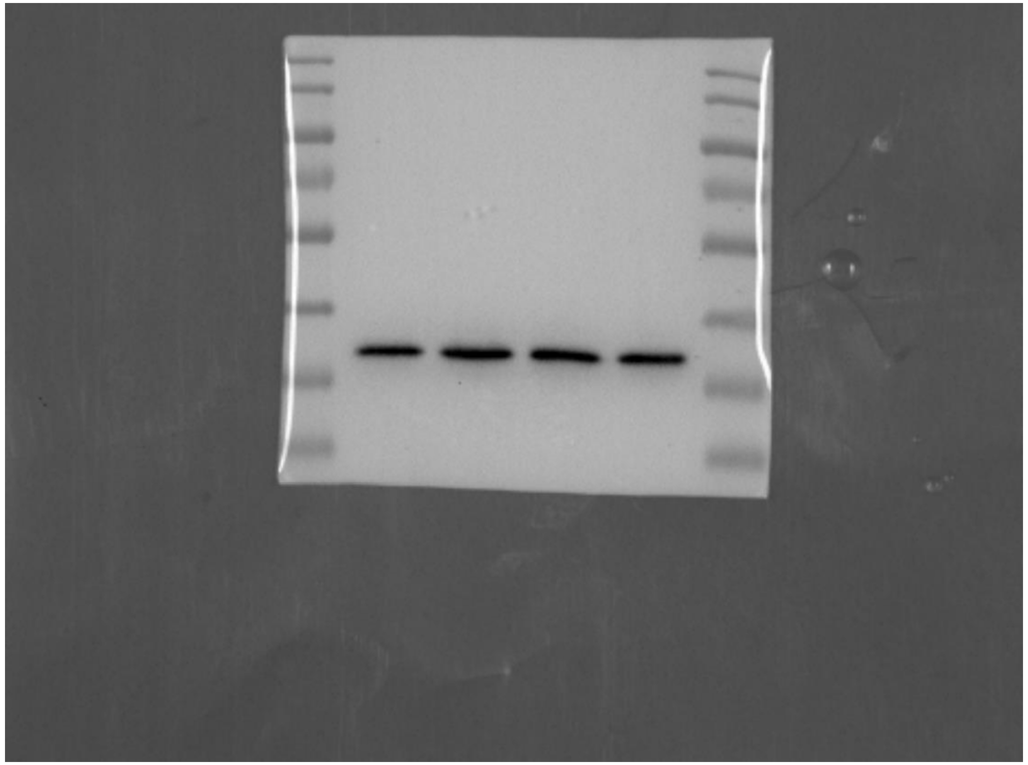

- **Figure 6C**

- MV4-11 FTO

Original

FTO

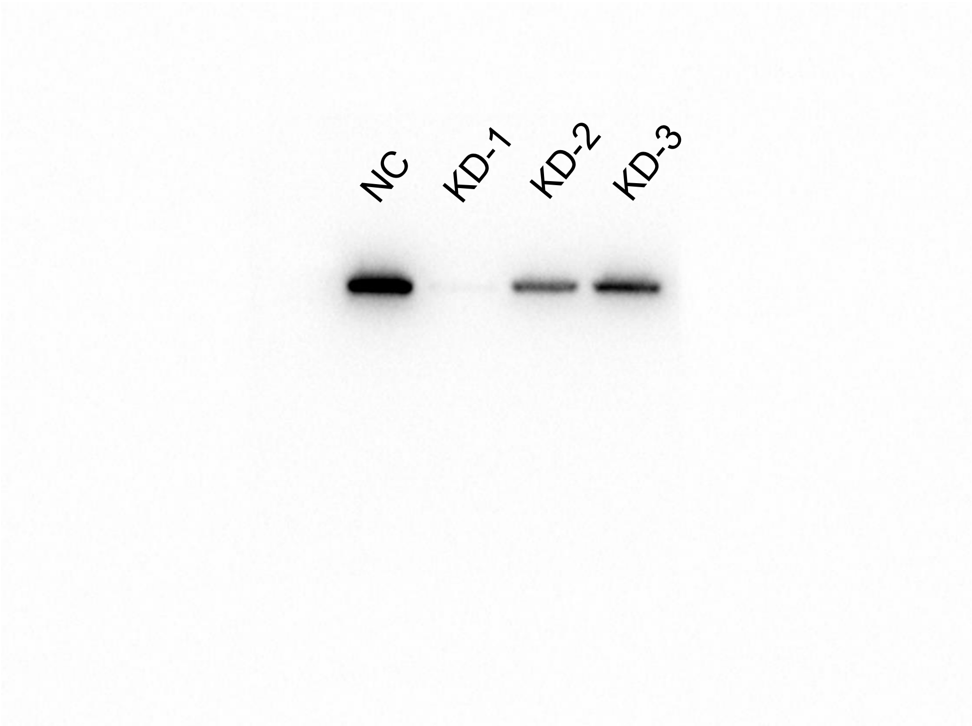

Merge

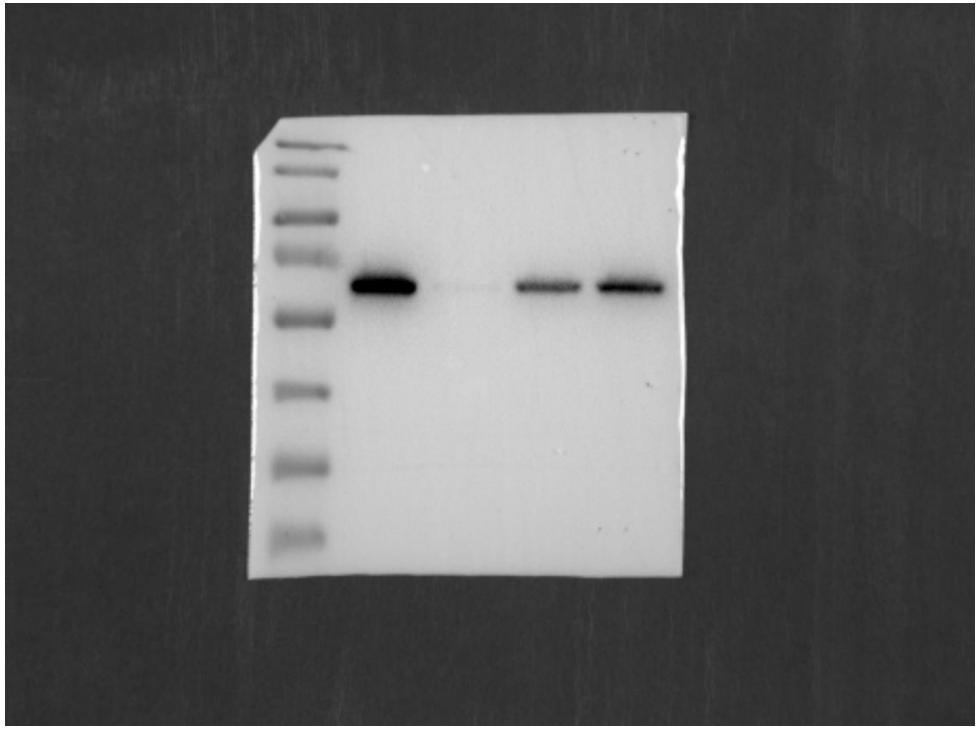

➢ **Figure 6C**

- MV4-11 FOXO3

Original

Merge

FOXO3

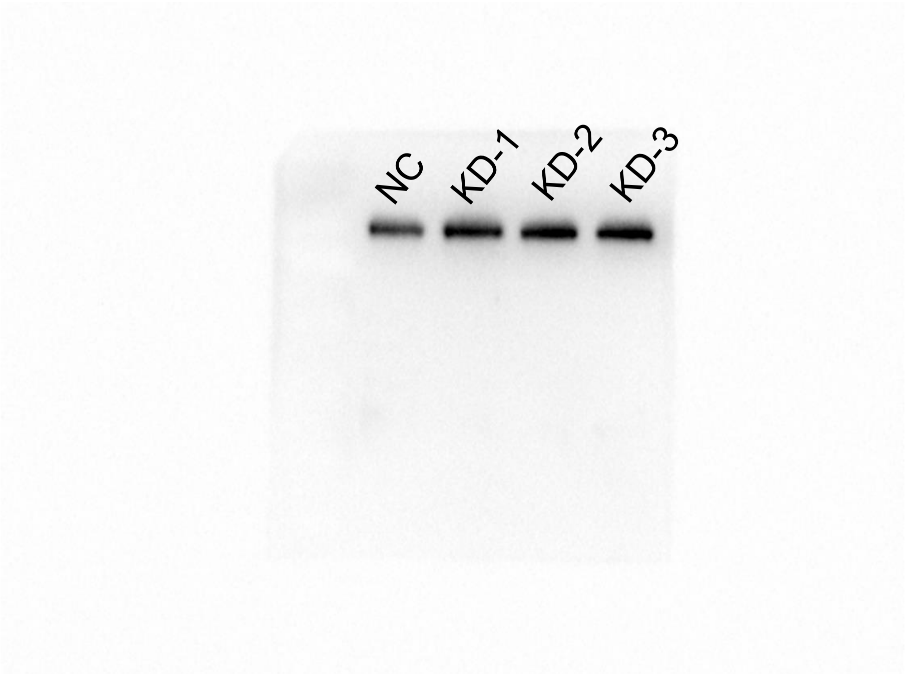

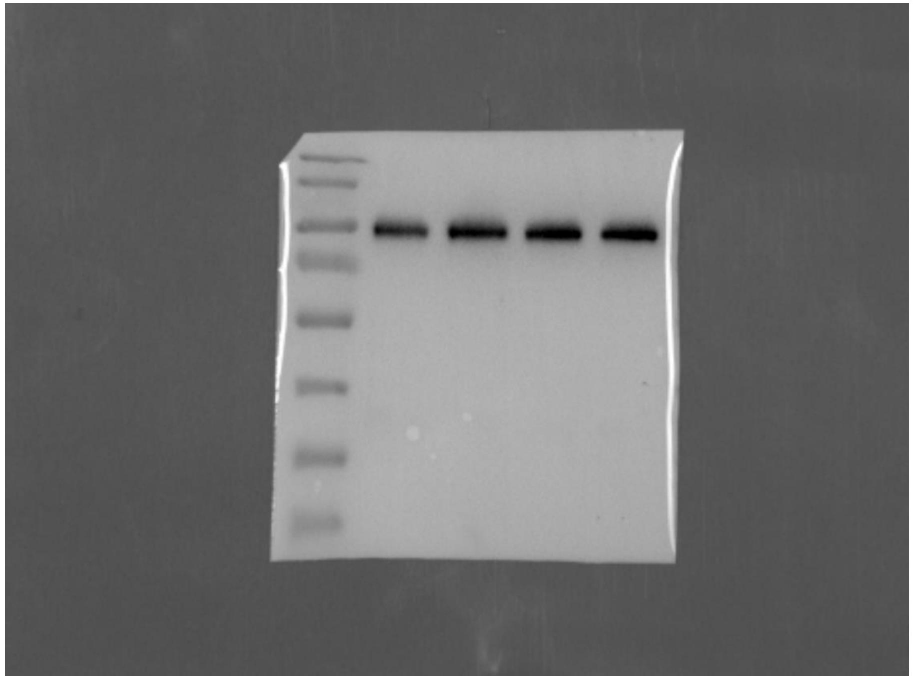

➢ **Figure 6C**

- MV4-11 GAPDH

Original

GAPDH

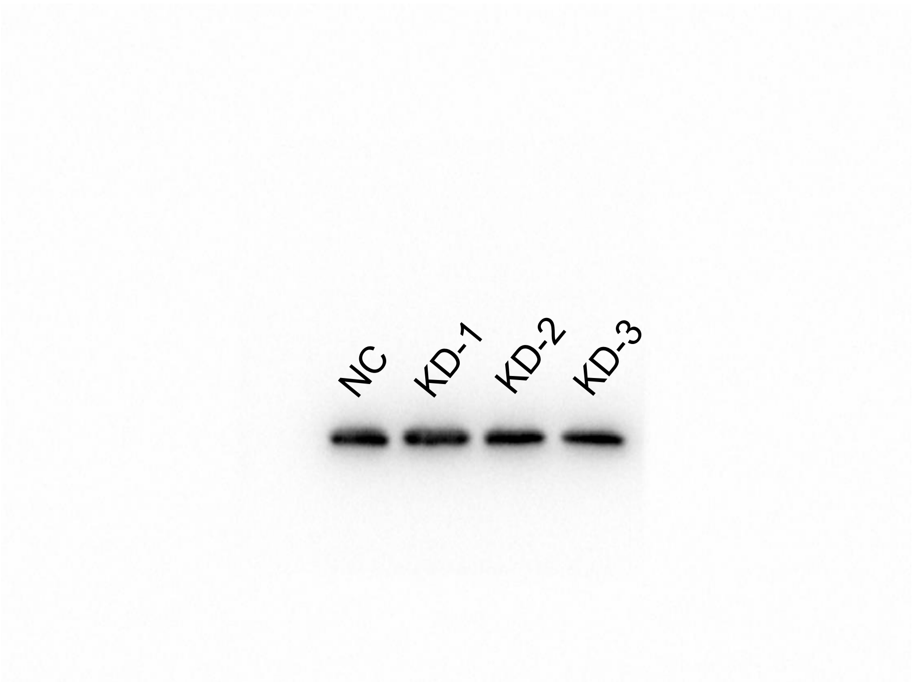

Merge

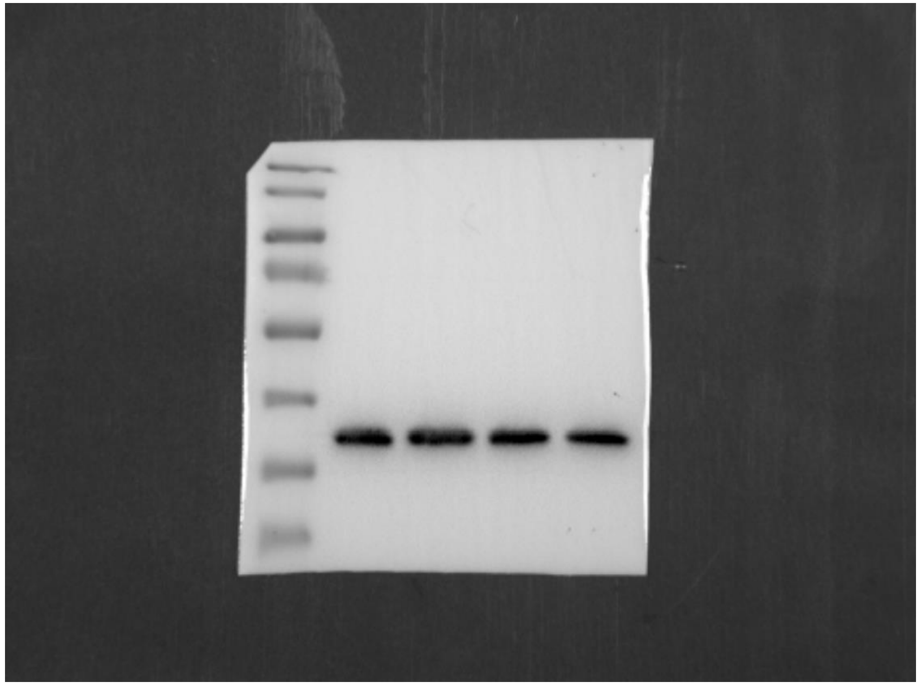

➢ **Figure 6C**

- THP-1 FTO

Original

FTO

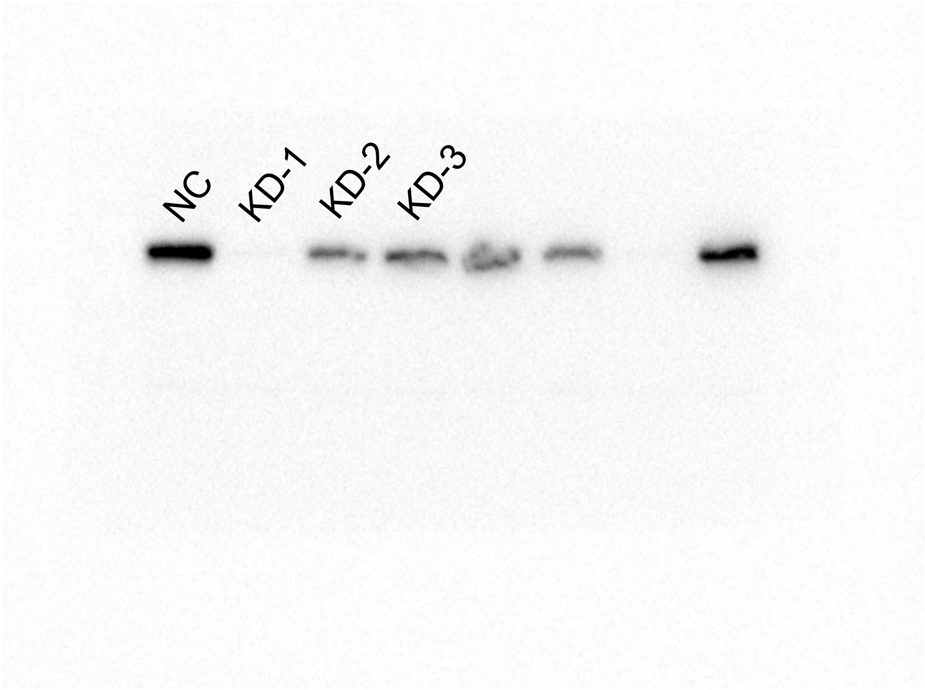

Merge

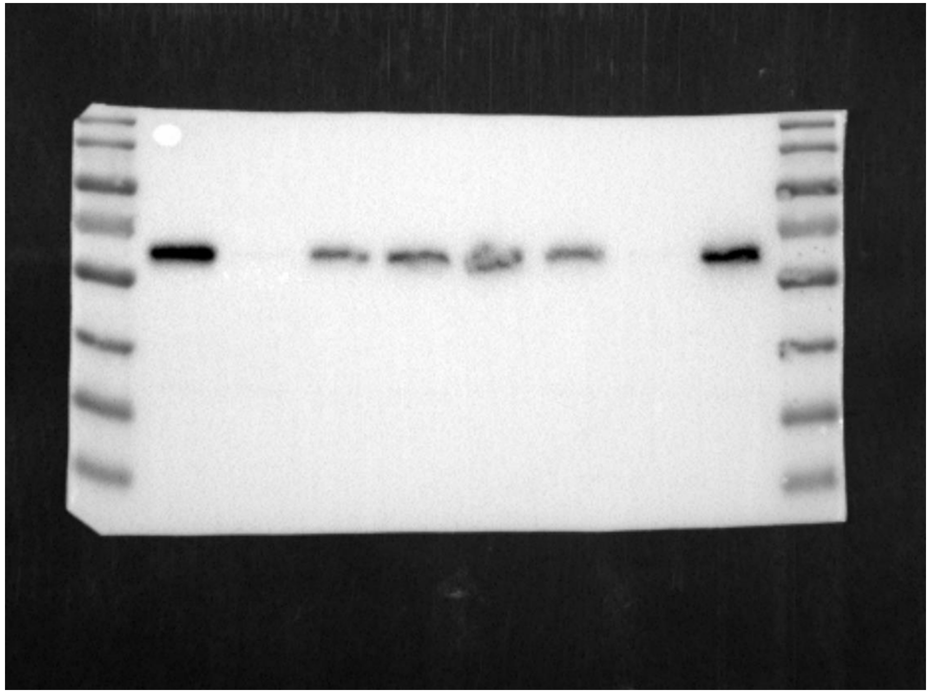

➢ **Figure 6C**

- THP-1 FOXO3

Original

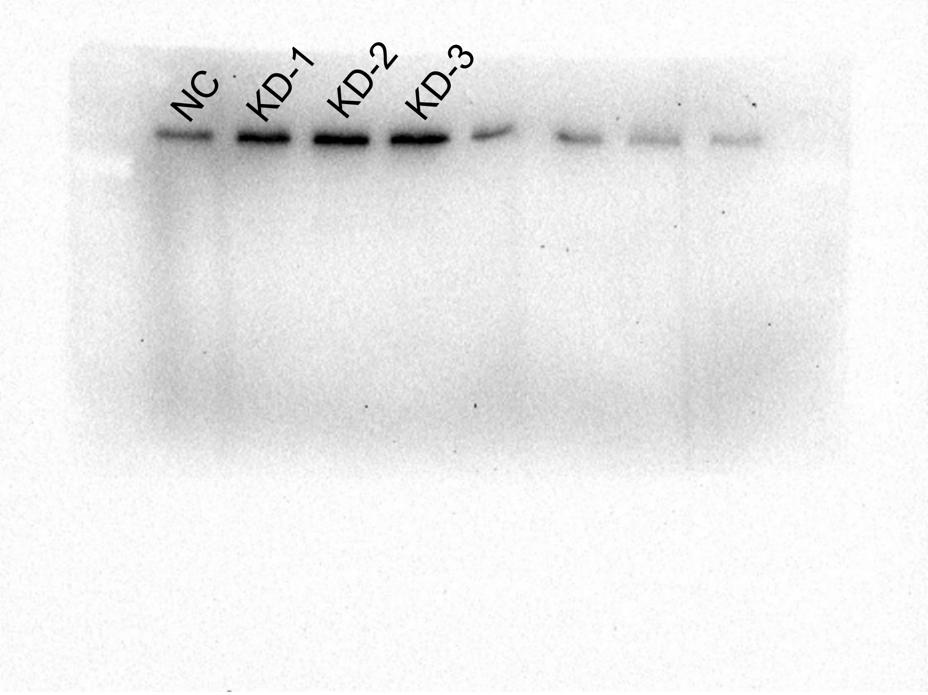

Merge

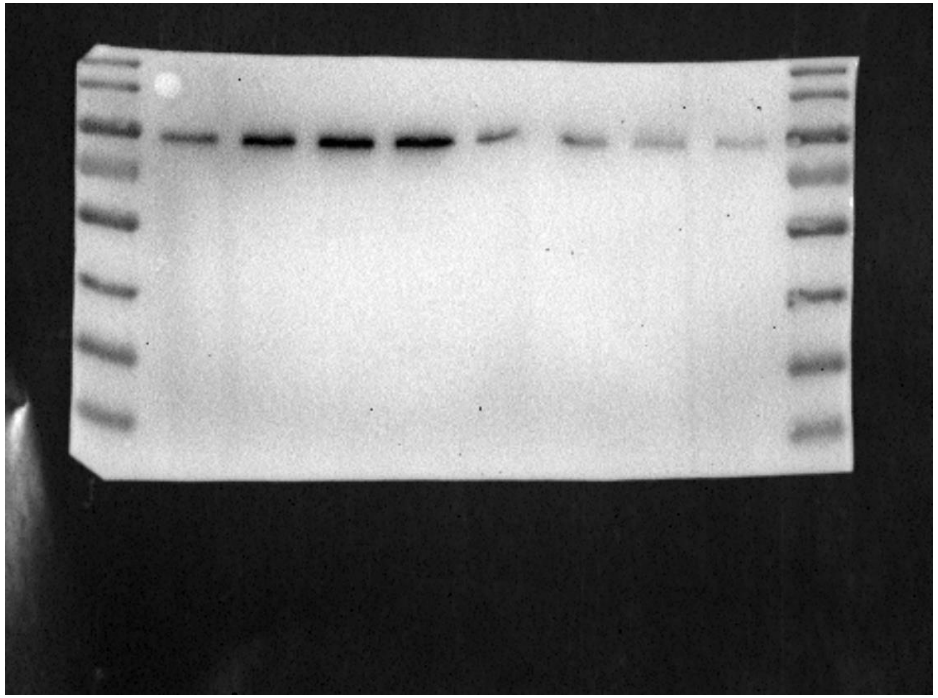

FOXO3

- **Figure 6C**

- THP-1 GAPDH

Original

GAPDH

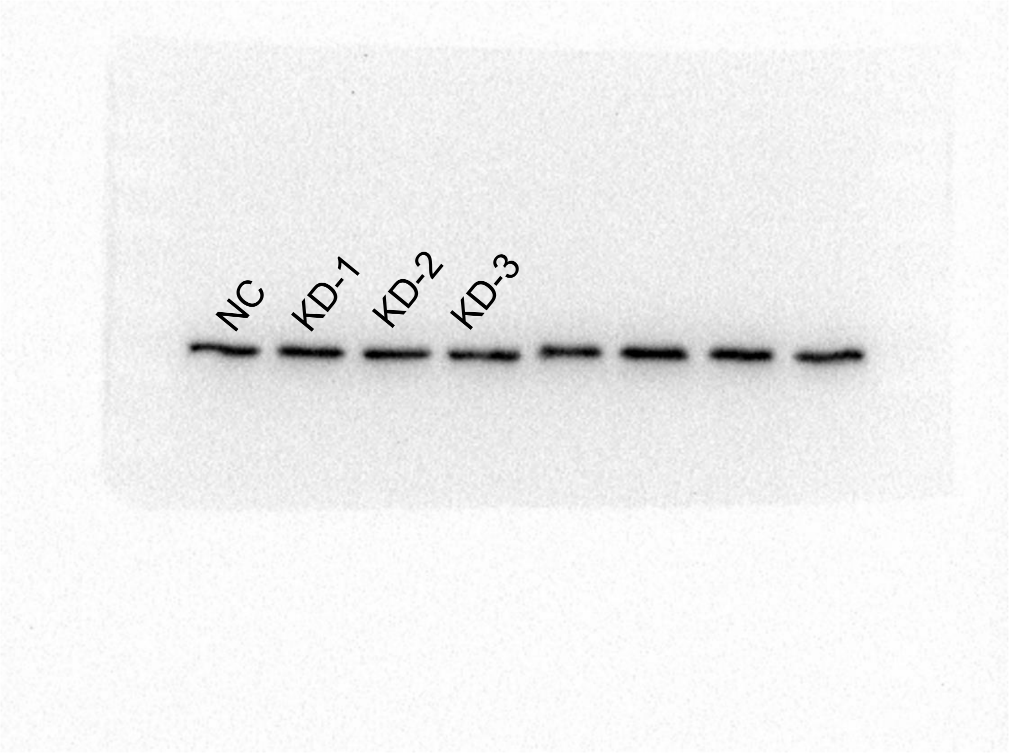

Merge

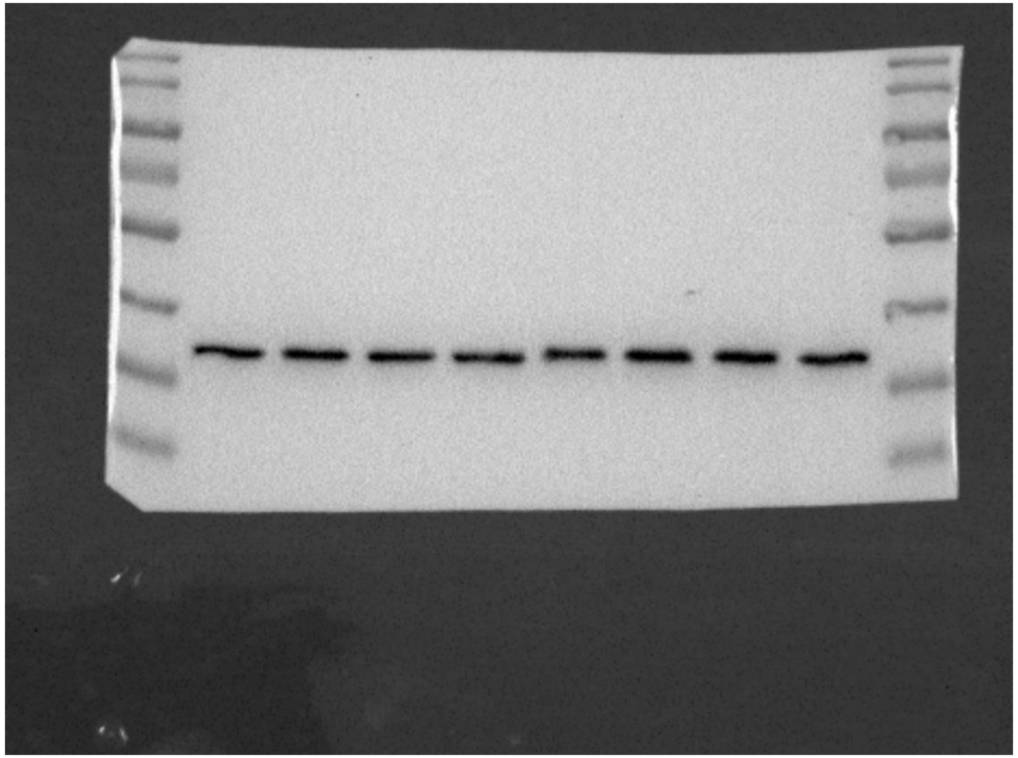

➢ **Figure S5D**

- FOXO3

Original

Merge

FOXO3

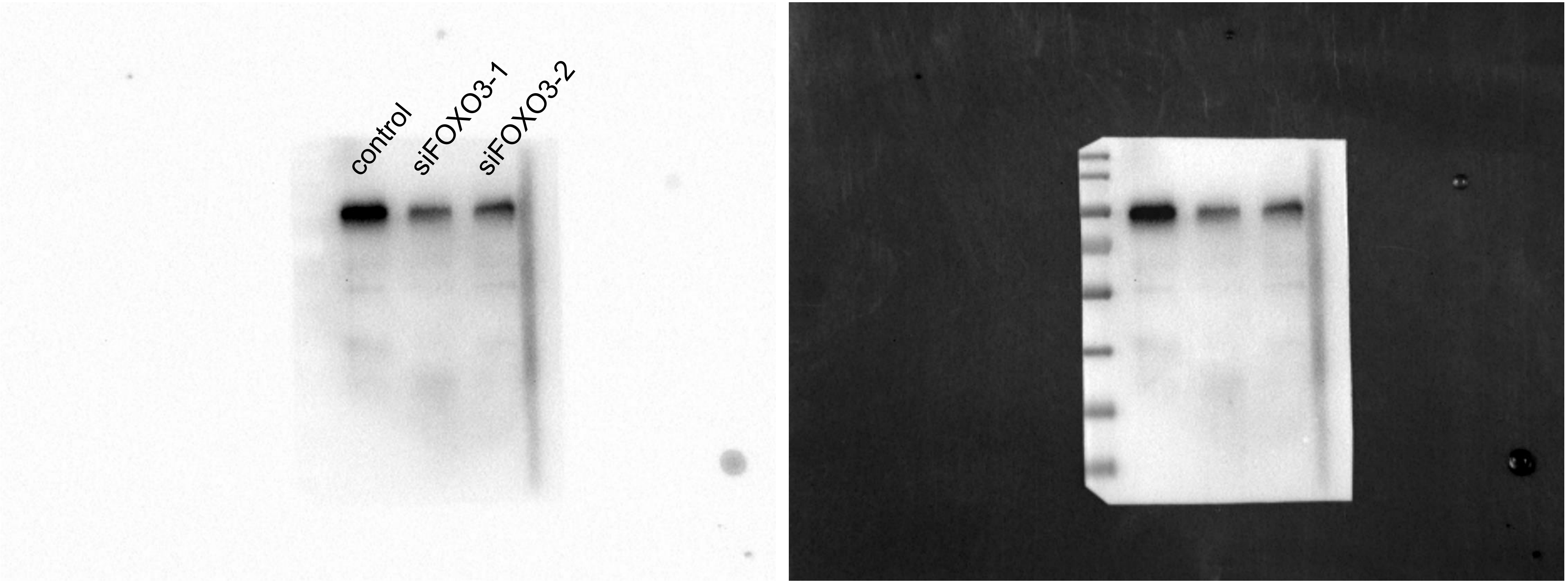

- **Figure S5D**

- GAPDH

Original

Merge

GAPDH

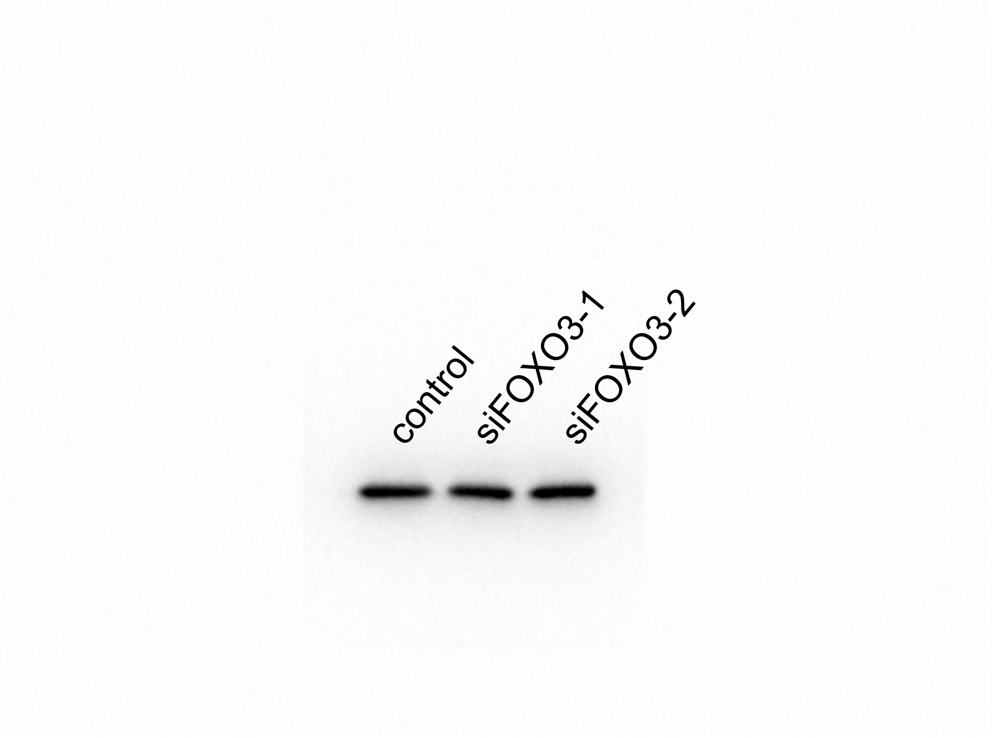

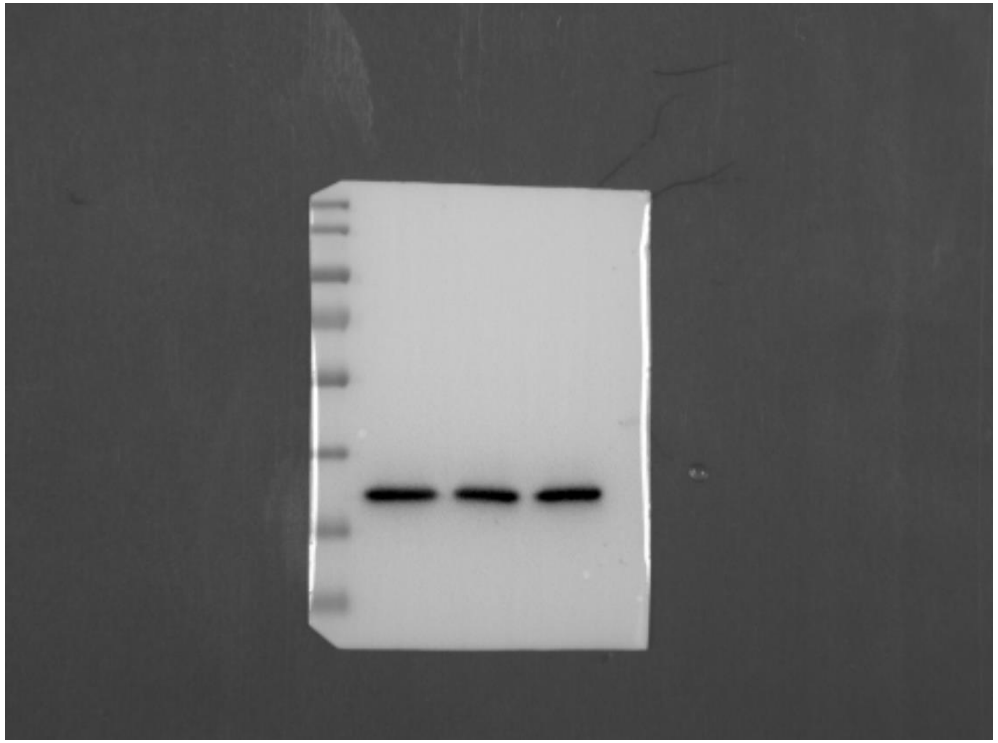

Supplement: Supplementary file 7 — Full and uncropped western blots [file 41420_2023_1505_MOESM7_ESM.pdf]
