# Supplementary material for: The role of m6A demethylase FTO in chemotherapy resistance mediating acute myeloid leukemia relapse
Source: Cell Death Discov. 2023 Jul 5;9:225. doi: 10.1038/s41420-023-01505-y (PMC10319875; doi:10.1038/s41420-023-01505-y)
Supplement: Supplementary file 8 — Key resources table [file 41420_2023_1505_MOESM8_ESM.doc]

Key Resources Table

| **ANTIBODIES** | | |
| --- | --- | --- |
| **REAGENT or RESOURCE** | **SOURCE** | **IDENTIFIER** |
| FTO | Abcam | 126606 |
| GAPDH | Cell Signaling Technology | 2118s |
| FOXO3 | Proteintech | 66428-1-lg |
| Anti-Mouse IgG(H+L) | Proteintech | SA00001-1 |
| Anti-Rabbit IgG(H+L) | Proteintech | SA00001-2 |
| PE anti-human CD11b Antibody | Biolegend | 301306 |
| APC anti-human CD14 Antibody | Biolegend | 367118 |
| **OLIGONUCLEOTIDES (5’-3’)** | | |
| **Primers** | | |
| *METTL3* F | CCCTATGGGACCCTGACAGA | |
| *METTL3* R | TGACACCAACCAAGCAGTGT | |
| *METTL14* F | GTTCGTAAGCTCCCGGTGAA | |
| *METTL14* R | ATCTCCTGCAAGCGGCTATC | |
| *WTAP* F | TGCTGTGTAAGGGCATTCGT | |
| *WTAP* R | TGTACTGGGCAAACTTGGCA | |
| *FTO* F | CTGGTTTGGCGATACCCCTT | |
| *FTO* R | CAGCCACTCAAACTCGACCT | |
| *ALKBH5* F | GCGACACTGTTGGCAATATGA | |
| *ALKBH5* R | TAGCAACCTGTCTTCACGCC | |
| *CREBBP* F | CCCTTAGTAACCAGGTGCGG | |
| *CREBBP* R | ATTGCACTCTGTTCGGGGTT | |
| *FOXO3* F | AGAAGTTCCCCAGCGACTTG | |
| *FOXO3* R | TCCCCACGTTCAAACCAACA | |
| *SIRPA* F | ATGAGCCCGAGAAGAATGCC | |
| *SIRPA* R | AGCATAGGTGAGGGTGTCCT | |
| *KMT2E* F | CAAAGTCTGAACAGCACGGC | |
| *KMT2E* R | CTGTTTGCTGGCTTGTGGTG | |
| *SYK* F | GGACTTTCCAAAGCACTGCG | |
| *SYK* R | CCCATCCGCTCTCCTTTCTC | |
| *POU2F2* F | TACCGTTGTCCCAAGCTTCC | |
| *POU2F2* R | GTAAGGGGCAGGGTTCCAC | |
| *BRD7* F | GGGAACGAAGTCACCGAACT | |
| *BRD7* R | GAGGCTTCTCAGGAGGCAAG | |
| *NDEL1* F | AGCTCGGGATGAGCTAGTTG | |
| *NDEL1* R | CCTTAATGGCCCGAGTCTGA | |
| *GLUC* F | CGACATTCCTGAGATTCCTGG | |
| *GLUC* R | TTGAGCAGGTCAGAACACTG | |
| *CLUC* F | GCTTCAACATCACCGTCATTG | |
| *CLUC* R | CACAGAGGCCAGAGATCATTC | |
| *GAPDH* F | GTCAAGGCTGAGAACGGGAA | |
| *GAPDH* R | AAATGAGCCCCAGCCTTCTC | |
| *18S* F | GGATGTAAAGGATGGAAAATACA | |
| *18S* R | TCCAGGTCTTCACGGAGCTTGTT | |
| **shRNAs** | | |
| KD-1 | GCTATTTCATGCTTGATGATC | |
| KD-2 | CGGTTCACAACCTCGGTTT | |
| **siRNAs** | | |
| si*FOXO3*-1 | GGACAAUAGCAACAAGUAUAC | |
| si*FOXO3*-2 | CUUGCUCAUAUCCCAUAUAAU | |

F: Forward primer

R: Reverse primer
